# Landscape of distant metastasis mode and current chemotherapy efficacy of the advanced biliary tract cancer in the United States, 2010‐2016

**DOI:** 10.1002/cam4.2794

**Published:** 2019-12-26

**Authors:** Jie Wang, Xiaobo Bo, Lingxi Nan, Chang Cheng Wang, Zhihui Gao, Tao Suo, Xiaoling Ni, Han Liu, Pinxiang Lu, Yueqi Wang, Houbao Liu

**Affiliations:** ^1^ Department of General Surgery Zhongshan Hospital Fudan University Shanghai China; ^2^ Biliary Tract Diseases Institute Fudan University Shanghai China; ^3^ Department of General Surgery Zhongshan‐Xuhui Hospital Affiliated to Fudan University Shanghai China

**Keywords:** biliary tract cancer, chemotherapy, distant metastasis, metastasis mode, PSM

## Abstract

**Background:**

The distant metastasis (DM) mode and treatment efficacies in the advanced biliary tract cancer (BTC) were obscure, and a credible evaluation is urgently needed.

**Method:**

A total of 6348 advanced BTC patients (ICC, intrahepatic cholangiocarcinoma, n = 1762; PHCC, perihilar cholangiocarcinoma, n = 1103; GBC, gallbladder cancer, n = 2580; DCC, distal cholangiocarcinoma, n = 538; AVC, carcinoma of Vater ampulla, n = 365) were enrolled from the Surveillance, Epidemiology, and End Results (SEER) database. Propensity score matching (PSM) process was carried out for less bias.

**Result:**

The proportion of M1 patients in each subtype at first diagnosis was 26.4% (ICC), 37.2% (PHCC), 41. 0% (GBC), 24.5% (DCC), and 12.7% (AVC), and the constitution of DM sites in different subtypes varied apparently. Moreover, the survival of metastasis sites was different (*P* < .05 in all the subtypes) where the multi‐metastasis and distant lymph node (dLN) only always indicated the worst and best prognosis, respectively. Chemotherapy presented the most significant survival impact with the lowest hazard ratio by multivariate cox model and still provided a survival improvement after PSM (all *P* < .001) in all subtypes. However, the median months manifested different between patients with and without chemotherapy among the subtypes (ICC, from 5 to 9; PHCC, from 6 to 10; AVC, from 4 to 9; GBC, from 6 to 7; DCC from 6 to 8).

**Conclusion:**

We provided a landscape about the detailed DM mode of the advanced BTC in a large population, found the survival differences among DM sites, and revealed the different chemotherapy efficacies in the BTC subtypes.

## Graphical abstract

Using a large population, we provided a detailed landscape about the DM mode of the advanced biliary tract cancer (BTC), found the survival differences among DM sites, and revealed the different chemotherapy efficacies in the BTC subtypes. The constitution and the survival of the DM sites varied significantly in different subtypes, and the chemotherapy efficacies manifested different among the subtypes.

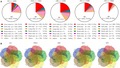

## 1 INTRODUCTION

Biliary system is a tree‐like network of tubular structures, and could be classified into five segments according to the anatomic position, which are intrahepatic bile duct, hilar bile duct, gallbladder, distal bile duct, and ampulla of Vater from distal to proximal, respectively. Correspondingly, biliary tract cancer (BTC), originated from biliary system, have five subtypes, including intrahepatic cholangiocarcinoma (ICC), perihilar cholangiocarcinoma (PHCC), gallbladder cancer (GBC), distal cholangiocarcinoma (DCC), and carcinoma of Vater ampulla (AVC).^1^ BTC is a kind of uncommon but relatively aggressive malignancy and made up about 4% of malignant digestive system tumors, with a gradually increasing incidence in the past years.^2^, ^3^

Complete surgical resection is the only curative treatment for BTC, but quite a few patients have no chance for radical surgery due to distant metastasis (DM) at initial diagnosis.^4^, ^5^ Although tremendous efforts have been made to explore the mechanisms of DM, the clinical features of DM in advanced BTC were poorly understood. Due to the low morbidity of BTC, the knowledge about the detailed DM mode mainly came from anatomy and clinical experience, and few clinical researches with large populations worked on it.^6^, ^7^

Proper treatments are vital for advanced BTC patients. According to the NCCN clinical practice guideline of hepatobiliary cancers, palliative surgery was not encouraged owing to the high risk and the limited benefit, and chemotherapy and radiation were recommended. Even though great progress on the treatments has been made, the survival time and life quality were still frustrating.^8^ To understand the DM mode and therapy efficacy of the advanced BTC, we analyzed the metastasis sites of each cancer subtype and assessed the efficacy of different treatments in a large population.

## 2 METHOD

### 2.1 Patients and data collection

Eligible patients were enrolled from the Surveillance, Epidemiology, and End Results (SEER) database of the US National Cancer Institute. The TNM information of the SEER cohort was obtained based on the following codes: Derived AJCC Stage Group, 7th ed (7th edition; 2010‐2015), Derived AJCC T (7th edition; 2010‐2015), Derived AJCC N (7th edition; 2010‐2015), Derived AJCC M (7th edition; 2010‐2015), Derived SEER Cmb Stg Grp (2016+), Derived SEER Combined T (2016+), Derived SEER Combined N (2016+), Derived SEER Combined M (2016+). The DM site was classified into liver only, distant lymph node (dLN) only, lung only, brain only, bone only, multi‐metastasis, and others, based on SEER combined mets at DX‐bone (2010+), SEER combined mets at DX‐brain (2010+), SEER combined mets at DX‐liver (2010+), SEER combined mets at DX‐lung (2010+), CS mets at DX (2004‐2015), and mets at DX‐distant LN (2016+). In the 7th AJCC staging system, “intrahepatic metastasis” was defined as T2b disease in ICC; however, these cases were recorded as liver only metastasis in the SEER database inconsequently, and we had to conduct analysis according to the record of SEER database.

Inclusion criteria included: (a) patients diagnosed with BTC between 2010 and 2016; (b) accurate tumor stage classification according to the 7th AJCC staging system; and (c) complete available follow‐up information. Patients whose BTC were not the first and only malignant primary tumor or who were lost to follow‐up were excluded in the cohort 1. Note that patients who died within 2 months after initial diagnosis confirmed or received radiation or palliative surgery were excluded in the propensity score matching (PSM) process for less bias in the cohort 2.

**Figure 1:**
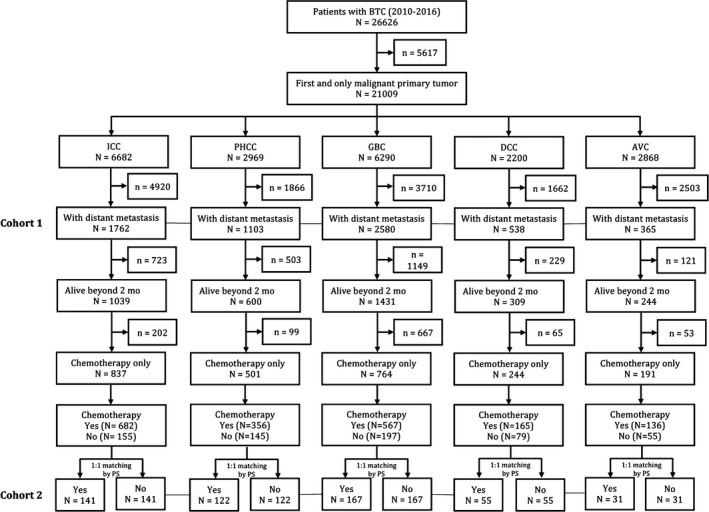
Flow chart of inclusion and exclusion in the study. The cohort 1 was for the analysis of distant metastasis mode and cox regression. The cohort 2 was for the survival analysis of chemotherapy efficacy and was built up by integrating the five subgroups. AVC, Carcinoma of Vater ampulla; BTC, biliary tract cancer DCC, Distal cholangiocarcinoma; GBC, Gallbladder cancer; ICC, Intrahepatic cholangiocarcinoma; PHCC, Perihilar cholangiocarcinoma; PS, Propensity score

The following clinicopathological variables were collected: clinic‐pathological factors (age at initial diagnosis, year at initial diagnosis, gender, race, tumor size, tumor differentiation, TNM stage, and metastasis site), economic factors (marital status, income, insurance, region, and residence city), and treatments (radiation, palliative surgery, and chemotherapy). Data regarding the detailed regimes of radiation, palliative surgery, and chemotherapy were unavailable. Survival duration was calculated from the date of initial diagnosis until the date of death or last follow‐up. The flow chart of inclusion and exclusion is shown in Figure 1.

### 2.2 Statistical analysis

Continuous variables were analyzed by Student's *t* test, and categorical variables were calculated by Chi‐squared test. Survival duration was calculated by the median overall survival (OS), and survival curves were constructed using the Kaplan‐Meier method. Analyses were performed using MedCalc 15.2.2 (MedCalc Software bvba, Ostend, Belgium) and GraphPad Prism 8.0.0 (GraphPad Software, San Diego, California USA, http://www.graphpad.com). PSM process was performed by MatchIt package in R version 3.4.0 (Bell Laboratories, Murray Hill, NJ). A two‐sided P‐value less than 0.05 was considered statistically significant.

## 3 RESULTS

### 3.1 Baseline characteristics

Our study included 6348 BTC patients with DM (ICC: 1762, PHCC: 1103, GBC: 2580, DCC: 538, AVC: 365) who were diagnosed between 2010 and 2016. As depicted in Table 1, most of the factors appeared to differ significantly among the five subtypes except residence, which suggested enormous diversity inside the BTC.

**Table 1:** Baseline characteristic of metastatic biliary tract cancer patients at initial diagnosis

Factors	ICC, n (%)	PHCC, n (%)	GBC, n (%)	DCC, n (%)	AVC, n (%)	*P*	Total, n (%)
Number of patients	1762	1103	2580	538	365		6348
Age, years							
≤59	573 (32.5)	280 (25.4)	625 (24.2)	127 (23.6)	89 (24.4)	**<.001**	1694 (26.7)
60‐69	577 (32.7)	364 (33.0)	751 (29.1)	184 (34.2)	99 (27.1)		1975 (31.1)
70‐79	417 (23.7)	287 (26.0)	688 (26.7)	133 (24.7)	103 (28.2)		1628 (25.6)
≥80	195 (11.1)	172 (15.6)	516 (20.0)	94 (17.5)	74 (20.3)		1051 (16.6)
Sex							
Male	924 (52.4)	537 (48.7)	791 (30.7)	276 (51.3)	200 (54.8)	**<.001**	2728 (43.0)
Female	838 (47.6)	566 (51.3)	1789 (69.3)	262 (48.7)	165 (45.2)		3620 (57.0)
Race							
White	1359 (77.1)	842 (76.3)	1900 (73.6)	415 (77.1)	283 (77.5)	**<.001**	4799 (75.6)
Black	144 (8.2)	104 (9.4)	381 (14.8)	58 (10.8)	46 (12.6)		733 (11.5)
Others	259 (14.7)	157 (14.2)	299 (11.6)	65 (12.1)	36 (9.9)		816 (12.9)
Year at diagnosis							
2010‐1013	787 (44.7)	577 (52.3)	1417 (54.9)	293 (54.5)	203 (55.6)	**<.001**	3277 (51.6)
2014‐2016	975 (55.3)	526 (47.7)	1163 (45.1)	245 (45.5)	162 (44.4)		3071 (48.4)
Marriage							
Yes	1031 (58.5)	588 (53.1)	1290 (50.0)	290 (53.9)	190 (52.1)	**<.001**	3389 (53.4)
No	731 (41.5)	515 (46.7)	1290 (50.0)	248 (46.1)	175 (47.9)		2959 (46.6)
Income							
Low	816 (46.3)	582 (52.8)	1327 (51.4)	289 (53.7)	187 (51.2)	**.001**	3201 (50.4)
High	946 (53.7)	521 (47.2)	1253 (48.6)	249 (46.3)	178 (48.8)		3147 (49.6)
Insurance							
Yes	1699 (96.4)	1043 (94.6)	2428 (94.1)	512 (95.2)	347 (95.1)	**.016**	6029 (95.0)
No	63 (3.6)	60 (5.4)	152 (5.9)	26 (4.8)	18 (4.9)		319 (5.0)
Region							
East	632 (35.9)	350 (31.7)	966 (37.4)	222 (41.3)	122 (33.4)	**<.001**	2292 (36.1)
Pacific Coast	922 (52.3)	646 (58.6)	1265 (49.0)	241 (44.8)	200 (54.8)		3274 (51.6)
Northern Plain	131 (7.4)	72 (6.5)	222 (8.6)	54 (10.0)	28 (7.7)		507 (7.99)
Alaska/Southwest	77 (4.4)	35 (3.2)	127 (4.9)	21 (3.9)	15 (4.1)		275 (4.33)
Residence city							
Small	262 (14.9)	158 (14.3)	404 (15.7)	107 (19.9)	57 (15.6)	.087	988 (15.6)
Middle	391 (22.2)	257 (23.3)	527 (20.4)	113 (21.0)	73 (20.0)		1361 (21.4)
Large	1109 (62.9)	688 (62.4)	1649 (63.9)	318 (59.1)	235 (64.4)		3999 (63.0)
Tumor size, mm							
≤30	115 (6.5)	165 (15.0)	394 (15.3)	118 (21.9)	123 (33.7)	**<.001**	915 (14.4)
30‐59	213 (12.1)	129 (11.7)	439 (17.0)	59 (11.00	61 (16.7)		901 (14.2)
>60	576 (32.7)	97 (8.8)	354 (13.7)	25 (4.6)	13 (3.6)		1065 (16.8)
Unknown	858 (48.7)	712 (64.5)	1393 (54.0)	336 (62.5)	168 (46.0)		3467 (54.6)
Differentiation							
I‐II	263 (14.9)	105 (9.5)	490 (19.0)	52 (9.7)	123 (33.7)	**<.001**	1033 (16.3)
III‐IV	277 (15.7)	135 (12.2)	686 (26.6)	75 (13.9)	103 (28.2)		1276 (20.1)
Unknown	1222 (69.4)	863 (78.3)	1404 (54.4)	411 (76.4)	139 (38.1)		4039 (63.6)
T stage							
T1‐T2	946 (53.7)	339 (30.7)	500 (19.4)	140 (26.0)	127 (34.8)	**<.001**	2052 (32.3)
T3‐T4	335 (19.0)	161 (14.6)	1340 (51.9)	183 (34.0)	140 (38.4)		2159 (34)
TX	481 (27.3)	603 (54.7)	740 (28.7)	215 (40.0)	98 (26.9)		2137 (33.7)
N stage							
N0	739 (41.9)	468 (42.4)	1073 (41.6)	272 (50.6)	176 (48.2)	**.003**	2728 (43.0)
N1	693 (39.3)	412 (37.4)	1034 (40.1)	175 (32.5)	134 (36.7)		2448 (38.6)
Nx	330 (18.7)	223 (20.2)	473 (18.3)	91 (16.9)	55 (15.1)		1172 (18.5)
Metastasis site							
Bone only	109 (6.2)	24 (2.2)	39 (1.5)	12 (2.2)	4 (1.1)	**<.001**	188 (3.0)
Brain only	2 (0.1)	1 (0.1)	3 (0.1)	1 (0.2)	3 (0.8)		10 (0.2)
Liver only	344 (19.5)	481 (43.6)	1281 (49.7)	248 (46.1)	200 (54.8)		2554 (40.2)
Lung only	159 (9.0)	51 (4.6)	79 (3.1)	31 (5.8)	33 (9.0)		353 (5.6)
dLN only	254 (14.4)	115 (10.4)	255 (9.9)	46 (8.6)	16 (4.4)		686 (10.8)
Other	288 (16.4)	158 (14.3)	463 (18.0)	96 (17.8)	54 (14.8)		1059 (16.7)
Multi‐metastasis	606 (34.4)	273 (24.8)	460 (17.8)	104 (19.3)	55 (15.1)		1498 (23.6)
Radiation							
Yes	193 (11.0)	89 (8.1)	193 (7.5)	46 (8.6)	29 (7.9)	**.002**	550 (8.7)
No	1569 (89.0)	1014 (91.9)	2387 (92.5)	492 (91.4)	336 (92.1)		5798 (91.3)
Palliative surgery							
Yes	71 (4.0)	50 (4.5)	858 (33.3)	43 (8.0)	50 (13.7)	**<.001**	1072 (16.9)
No	1691 (96.0)	1053 (95.5)	1722 (66.7)	495 (92.1)	315 (86.3)		5276 (83.1)
Chemotherapy							
Yes	1049 (59.5)	527 (47.8)	1283 (49.7)	261 (48.5)	212 (58.1)	**<.001**	3332 (52.5)
No	713 (40.5)	576 (52.2)	1297 (50.3)	277 (51.5)	153 (41.9)		3016 (47.5)

Note

*P* < .05 is considered statistically significant (bold).

Abbreviation: AVC, Carcinoma of Vater ampulla; dLN, Distant lymph node; DCC, Distal cholangiocarcinoma; GBC, Gallbladder cancer; ICC, Intrahepatic cholangiocarcinoma; PHCC, Perihilar cholangiocarcinoma.

### 3.2 Distant metastasis mode

**Figure 2:**
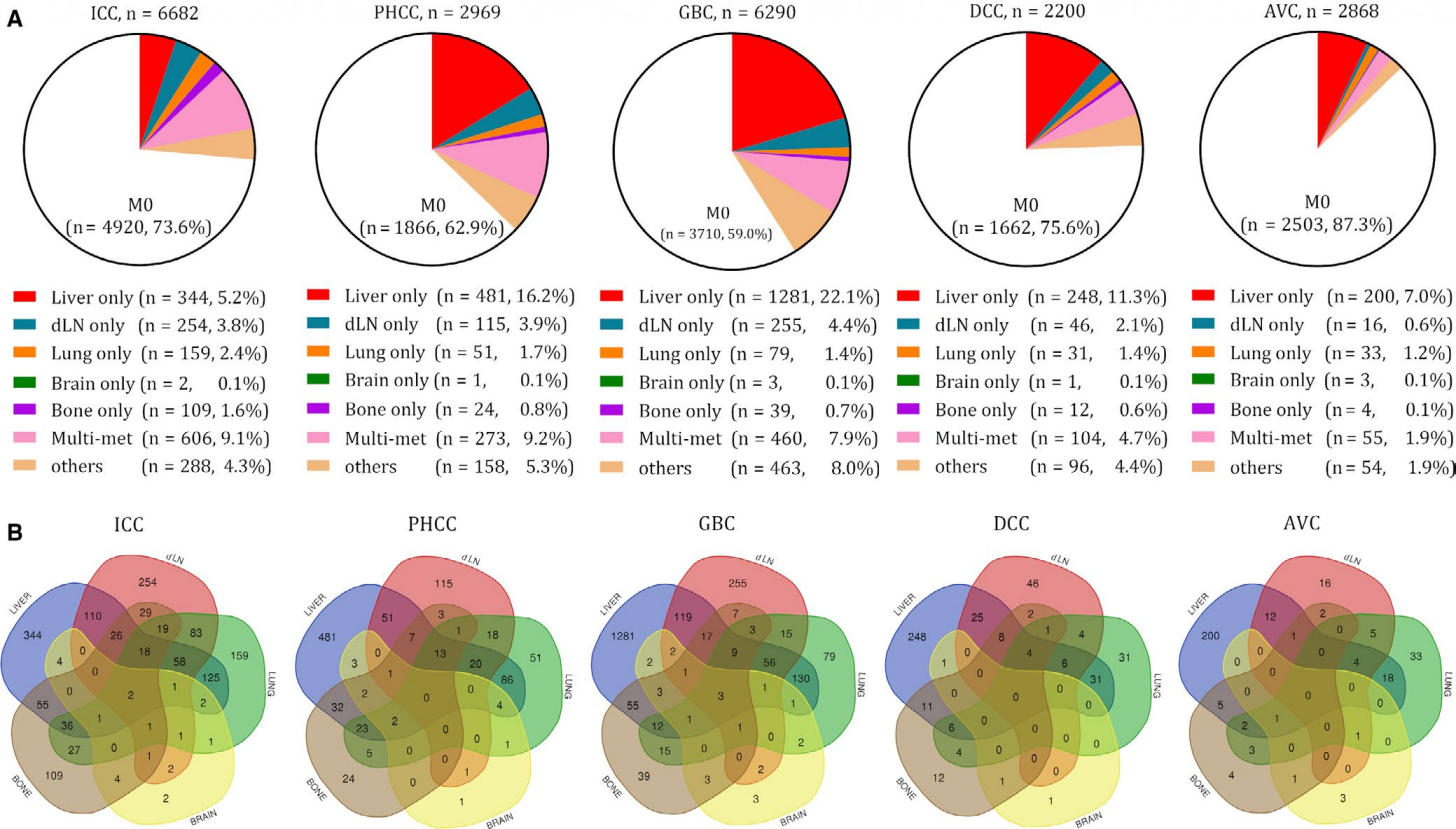
Distant metastasis mode of each subtype of the BTC. A, proportion of the M1 patients at first diagnosis in each subtype. B, Venn diagrams of the distribution of distant metastatic sites in each subtype. BTC, biliary tract cancer; ICC, Intrahepatic cholangiocarcinoma; PHCC, Perihilar cholangiocarcinoma; GBC, Gallbladder cancer; DCC, Distal cholangiocarcinoma; AVC, Carcinoma of Vater ampulla; dLN, Distant lymph node

DM mode was an important form of the diversity. As shown in Figure 2A, the proportion of M1 patients in each subtype at first diagnosis was 26.4% in the ICC, 37.2% in the PHCC, 41. 0% in the GBC, 24.5% in the DCC, and 12.7% in the AVC, respectively. The GBC had the highest DM rate and the highest liver only rate (22.1%), which meant that more than 2/5 of the GBC patients had the DM, and over half of them were the liver only DM. The DM rate and the liver only rate of the PHCC were slightly less than that in the GBC, while its multi‐metastasis rate was the highest. Although the ICC and the DCC had similar DM rate (more than 1/3), the most common DM site of the ICC and the DCC were the multi‐metastasis (9.1%) and the liver only (11.3%), respectively. Conversely, only less than 1/5 of the M1 ICC patients had the liver only DM, and nearly half of the M1 DCC patients had the liver only DM. More than 1/3 of the M1 DCC patients had the multi‐metastasis, while only 1/6 of the M1 ICC patients had the multi‐metastasis. The AVC had the lowest DM rate (12.7%), and nearly half of them were liver only DM.

As other metastasis sites, the dLN only was commonly seen in the ICC (3.8%), the PHCC (3.9%), and the GBC (4.4%); the bone only was mostly found in the ICC (1.6%); the lung only rate was similar in all the subtypes (1.2%‐2.4%), and the brain only was extremely rare in all the subtypes (all ≤0.1%). In addition, there were still part of patients without accurate record of the metastasis sites in all the subtypes.

The multi‐metastasis could be found in some patients at initial diagnosis. The most common combination was liver plus lung, and the second one was liver plus dLN. This was in accord with the fact that liver, dLN, and lung were the top three DM sites in most of the BTC (Figure2B and Figure S2‐S7), indicating that the combinations of the multi‐metastasis were closely related to the DM rate of a single site. However, although the rate of liver plus lung was higher than that of liver plus dLN (liver plus lung rate vs liver plus dLN rate: ICC, 7.1% vs 6.2%; PHCC, 7.8% vs 4.6%; GBC, 5.0% vs 4.6%; DCC, 5.8% vs 4.7%; AVC, 4.9% vs 3.3%), the dLN DM rate was higher than the lung DM rate in most of the BTC (dLN vs lung: ICC, 14.4% vs 9.0%; PHCC, 10.4% vs 4.6%; GBC, 9.9% vs 3.1%; DCC, 8.6% vs 5.8%; AVC, 4.4% vs 9.0%) (Figure 2B, Figure S2‐S7), suggesting that the multi‐site metastasis might be progressed step by step but not randomly combined. All the specific combinations of different sites are shown in Figure 2B, and more detailed information about metastasis mode is shown in Figure S2‐S7.

**Figure 3:**
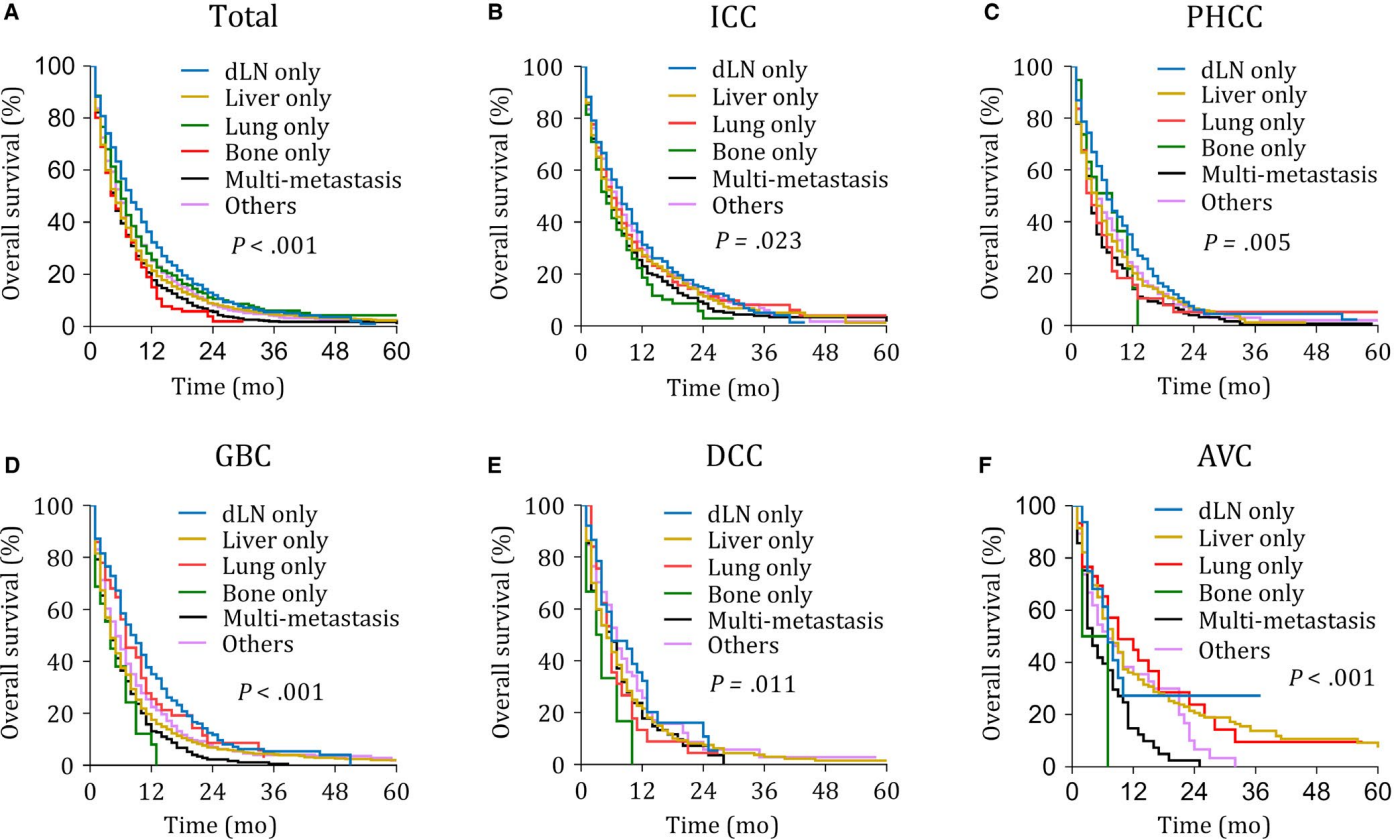
Kaplan–Meier curves of overall survival according to metastasis sites in the total cohort and each subtype. AVC, Carcinoma of Vater ampulla; BTC, biliary tract cancer; DCC, Distal cholangiocarcinoma; dLN, Distant lymph node; GBC, Gallbladder cancer; ICC, Intrahepatic cholangiocarcinoma; PHCC, Perihilar cholangiocarcinoma

Although all the M1 BTC patients shared a compromised prognosis, survival of different metastasis sites displayed significantly different (*P* < .001 in the ICC, *P* = .005 in the PHCC, *P* < .001 in the GBC, *P* = .011 in the DCC, and *P* < .001 in the AVC, respectively) (Figure 3). In our survival analysis, multi‐metastasis always indicated a shortest survival, while dLN only always indicated a better prognosis. Moreover, the bone only in the ICC, GBC, and DCC, the lung only in the PHCC, and the liver only in the GBC were also poor predictors for OS. The brain only was excluded in this analysis for the insufficient patient numbers.

### 3.3 Univariate and multivariate cox analysis

Univariate and multivariate cox analysis were conducted to further identify the potential factors that might influence survival besides metastasis sites. In the univariate cox analysis, age, race, marital status, insurance, tumor size, differentiation, T stage, N stage, metastasis sites, and treatments (radiation, palliative surgery, and chemotherapy) were identified to be significantly associated with OS in at least two subtypes (Table S1). Besides, sex in the ICC, and year at diagnosis and residence in the GBC were also significant predictors.

These factors proved to be significant predictors above were then entered into the multivariate cox proportional hazards model of different subtypes separately. As shown in Table 2, the independent prognostic indicators were demonstrated in the ICC (age, sex, differentiation, metastasis site, radiation, palliative surgery, and chemotherapy), the PHCC (age, race, region, tumor size, metastasis site, palliative surgery, and chemotherapy), the GBC (age, income, tumor size, differentiation, N stage, metastasis site, palliative surgery, and chemotherapy), the DCC (palliative surgery and chemotherapy), and the AVC(differentiation, metastasis site, palliative surgery, and chemotherapy). Based on the results, the clinic‐pathological factors (age, race, sex, differentiation, tumor size, and metastasis site) and the treatments (palliative surgery and chemotherapy) play important roles in patient survival, while the economic factors' (region and income) influence was rather limited. Interestingly, T stage and N stage also only had limited influence. Furthermore, factors identified above in the ICC, PHCC, and GBC were more abundant compared with that in the DCC and the AVC, where the treatments had the most significant influence in patient prognosis.

**Table 2:** Multivariate cox regression models of prognostic factors for overall survival

Factors	ICC (n = 1762)		PHCC (n = 1103)		GBC (n = 2580)		DBDC (n = 538)		AVC (n = 365)	
	HR (95% CI)	*P*	HR (95% CI)	*P*	HR (95% CI)	*P*	HR (95% CI)	*P*	HR (95% CI)	*P*
Age, years										
60‐69 vs ≤ 59	1.143 (1.004‐1.302)	**.045**	1.268 (1.071‐1.502)	**.006**	1.091 (0.971‐1.226)	0.147	0.906 (0.704‐ 1.165)	.443	0.868 (0.618‐1.218)	.415
70‐79 vs ≤ 59	1.270 (1.099‐1.466)	**.001**	1.374 (1.146‐1.647)	**<.001**	1.177 (1.044‐1.326)	**.008**	1.258 (0.963‐1.645)	.095	1.152 (0.826‐1.607)	.406
≥80 vs ≤ 59	1.410 (1.168‐1.703)	**<.001**	1.226 (0.991‐1.517)	.063	1.422 (1.246‐1.622)	**<.001**	1.299 (0.961‐1.755)	.091	0.974 (0.662‐1.432)	.892
Sex										
Male vs Female	0.772 (0.694‐0.858)	**<.001**								
Race										
Black vs White			0.919 (0.738‐1.145)	.456			1.105 (0.818‐1.492)	.517		
Others vs White			0.661 (0.546‐0.802)	**<.001**			0.846 (0.630‐1.136)	.268		
Year at diagnosis										
14‐16 vs 10‐13					0.958 (0.878‐1.045)	.334				
Marriage										
Yes vs No	1.106 (0.993‐1.233)	.069			1.054 (0.967‐1.149)	.237	1.179 (0.977‐1.423)	.088		
Income										
Low vs High					1.155 (1.062‐1.256)	**<.001**				
Insurance										
No vs Yes	1.001 (0.759‐1.322)	.993			0.949 (0.793‐1.135)	.566				
Region										
Pacific Coast vs East	1.142 (0.934‐1.396)	.197	0.724 (0.555‐0.943)	**.016**						
Northern Plain vs East	0.972 (0.868‐1.089)	.629	0.908 (0.786‐1.049)	.193						
Alaska/Southwest vs East	0.910 (0.701‐1.183)	.484	0.926 (0.632‐1.338)	.663						
Residence city										
Middle vs Large					1.018 (0.915‐1.132)	.745				
Small vs Large					1.136 (0.947‐1.209)	.281				
Tumor size, mm										
30‐59 vs ≤ 29	0.871 (0.672‐1.128)	.298	1.104 (0.863‐1.412)	.435	1.115 (0.967‐1.149)	.168	1.155 (0.822‐1.622)	.410		
>60 vs ≤ 29	0.985 (0.783‐1.240)	.899	1.363 (1.037‐1.791)	**.017**	1.243 (1.056‐1.462)	**.009**	1.010 (0.621‐1.642)	.969		
Unknown vs ≤ 29	1.023 (0.815‐1.285)	.844	1.252 (1.042‐1.504)	**.017**	1.276 (1.119‐1.456)	**<.001**	1.099 (0.862‐1.401)	.449		
Differentiation										
III/ IV vs I‐II	1.312 (1.085‐1.588)	**.005**			1.460 (1.282‐1.663)	**<.001**	1.167 (0.778‐1.749)	.458	1.816 (1.333‐2.474)	**<.001**
Unknown vs I‐II	1.373 (1.178‐1.601)	**<.001**			1.242 (1.081‐1.426)	**.002**	1.137 (0.803‐1.609)	.471	1.122 (0.849‐1.484)	.421
T stage										
T3‐T4 vs T1‐T2	0.948 (0.825‐1.089)	.448			0.955 (0.849‐1.075)	.447				
TX vs T1‐T2	0.905 (0.790‐1.037)	.154			0.940 (0.812‐1.087)	.406				
N stage										
N1 vs N0			1.001 (0.860‐1.165)	.990	1.024 (0.928‐1.130)	.635				
Nx vs N0			1.169 (0.984‐1.389)	.107	1.129 (1.001‐1.273)	**.049**				
Metastasis site										
Liver vs DLN	0.970 (0.803‐1.171)	.751	1.243 (0.986‐ 1.569)	.068	1.348 (1.153‐1.575)	**<.001**	0.968 (0.680‐1.378)	.856	0.840 (0.452‐1.559)	.582
Lung vs DLN	1.113 (0.895‐1.383)	.338	1.346 (0.950‐1.908)	.097	1.022 (0.771‐1.355)	.882	1.047 (0.635‐1.725)	.859	0.789 (0.388‐1.606)	.515
Brain vs DLN	3.075 (0.761‐12.434)	.117	2.027 (0.274‐14.979)	.491	1.419 (0.453‐4.447)	.551	5.244 (0.710‐38.728)	.106	2.127 (0.578‐7.833)	.259
Bone vs DLN	1.323 (1.030‐1.700)	**.029**	1.089 (0.673‐1.762)	.731	1.901 (1.306‐2.766)	**<.001**	1.594 (0.811‐3.133)	.178	3.499 (1.096‐11.167)	**.035**
Others vs DLN	1.079 (0.894‐1.303)	.432	1.121 (0.859‐1.463)	.403	1.307 (1.097‐1.558)	**<.001**	0.801 (0.539‐1.190)	.274	0.934 (0.473‐1.844)	.844
Multi vs DLN	1.332 (1.126‐1.576)	**<.001**	1.612 (1.266‐2.053)	**<.001**	1.470 (1.236‐1.748)	**<.001**	1.204 (0.820‐1.768)	.346	1.683 (0.869‐3.260)	.125
Radiation										
Yes vs No	0.750 (0.631‐0.891)	**.001**	0.895 (0.704‐1.138)	.368	0.950 (0.804‐1.123)	.553				
Palliative surgery										
Yes vs No	0.528 (0.398‐0.699)	**<.001**	0.402 (0.290‐0.558)	**<.001**	0.636 (0.561‐0.720)	**<.001**	0.452 (0.286‐0.714)	**<.001**	0.356 (0.237‐0.534)	**<.001**
Chemotherapy										
Yes vs No	0.274 (0.243‐0.310)	**<.001**	0.381 (0.330‐0.438)	**<.001**	0.403 (0.367‐0.443)	**<.001**	0.350 (0.285‐0.429)	**<.001**	0.309 (0.234‐0.409)	**<.001**

Note

*P*<0.05 is considered statistically significant (bold).

Abbreviations:AVC, Carcinoma of Vater ampulla; CI, Confidence interval; dLN, Distant lymph node; GBC, Gallbladder cancer; DCC, Distal cholangiocarcinoma; HR, Hazard ratio; ICC, Intrahepatic cholangiocarcinoma; PHCC, Perihilar cholangiocarcinoma.

### 3.4 Chemotherapy efficacy in different BTC subtypes

Treatments for the M1 BTC patients included radiation, palliative surgery, and chemotherapy. Radiation only worked in the ICC with a higher hazard ratio (HR) than chemotherapy (Table 2), and palliative surgery was always considered harmful in the NCCN clinical practice guideline of hepatobiliary cancers, and only very few patients received it (Table 1). Chemotherapy was the main choice at present, and it proved to be a significant method to prolong patient survival in multivariate cox model (yes vs no: ICC, HR (95%CI): 0.274 (0.243‐0.310), *P* < .001; PHCC, HR (95%CI): 0.381 (0.330‐0.438), *P* < .001; GBC, HR (95%CI): 0.403 (0.367‐0.443), *P* < .001; DCC, (95%CI): 0.350 (0.285‐0.429), *P* < .001; AVC, HR (95%CI), 0.309 (0.234‐0.409), *P* < .001).

To further assess the therapeutic value of chemotherapy, Kaplan‐Meier survival analysis was adopted and median survival was calculated simultaneously. Patients lived less than 2 months or received radiation or palliative surgery were excluded in this analysis to reduce possible bias between chemotherapy group and no chemotherapy group. However, significant difference of baseline characteristics existed between two groups in the total cohort (Table 3) and all the subtypes (Table S2‐S6). PSM was conducted to correct bias in each subtype separately with an appropriate caliper of 0.04, and then all the baseline characteristics between two groups presented no difference in the five subtypes (Table S2‐S6). The total cohort was integrated from the five subtypes (Table 3).

**Table 3:** Baseline characteristics of the without (No) and with chemotherapy (Yes) groups before and after PSM

Factors	Before PSM			After PSM		
	No (n = 631)	Yes (n = 1906)	*P*	No (n = 516)	Yes (n = 516)	*P*
Location						
ICC	155	682	**<.001**	141	141	1.000
PHCC	145	356		122	122	
GBC	197	567		167	167	
DCC	79	165		55	55	
AVC	55	136		31	31	
Age, years						
≤59	132	675	**<.001**	125	112	.499
60‐69	163	655		154	169	
70‐79	159	451		136	167	
≥80	177	125		101	68	
Sex						
Male	262	856	.149	226	240	.416
Female	369	1050		290	276	
Race						
White	455	1453	**.011**	379	379	.669
Black	72	219		58	68	
Others	104	234		79	69	
Year at diagnosis						
2010‐1013	361	994	**.031**	293	286	.707
2014‐2016	270	912		223	230	
Marriage						
Yes	266	1172	**<.001**	246	259	.455
No	365	734		270	257	
Income						
Low income	308	990	0.187	255	256	1.000
High income	323	916		261	260	
Insurance						
Yes	594	1851	**<.001**	17	25	.271
No	37	55		499	491	
Region						
East	190	738	**<.001**	165	195	.209
Pacific Coast	48	152		35	36	
Northern Plain	365	946		293	268	
Alaska/Southwest	28	70		23	17	
Residence city						
Small	420	1214	.327	348	320	.168
Middle	129	403		102	114	
Large	82	289		66	82	
Tumor size, mm						
≤30	90	247	.602	72	77	.912
30‐59	93	272		76	67	
>60	75	369		69	76	
Unknown	373	1018		299	296	
Differentiation						
I‐II	92	271	.474	74	65	.965
III‐IV	76	289		59	78	
Unknown	463	1346		383	373	
T stage						
T1‐T2	186	661	**.001**	157	158	.981
T3‐T4	183	595		154	156	
TX	262	650		205	202	
N stage						
N0	284	786	.336	238	229	.839
N1	199	807		168	191	
Nx	148	313		110	96	
Metastasis site						
dLN only	68	243	**<.001**	56	68	.515
Liver only	294	677		227	217	
Lung only	44	132		33	26	
Brain only	2	0		2	0	
Bone only	15	37		13	5	
Other	115	305		100	80	
Multi‐metastasis	93	512		85	120	

Note

*P* < .05 is considered statistically (bold).

Abbreviations: AVC, Carcinoma of Vater ampulla; dLN, Distant lymph node; DBDC, Distal cholangiocarcinoma; GBC, Gallbladder cancer; ICC, Intrahepatic cholangiocarcinoma; PHCC, Perihilar cholangiocarcinoma.

**Figure 4:**
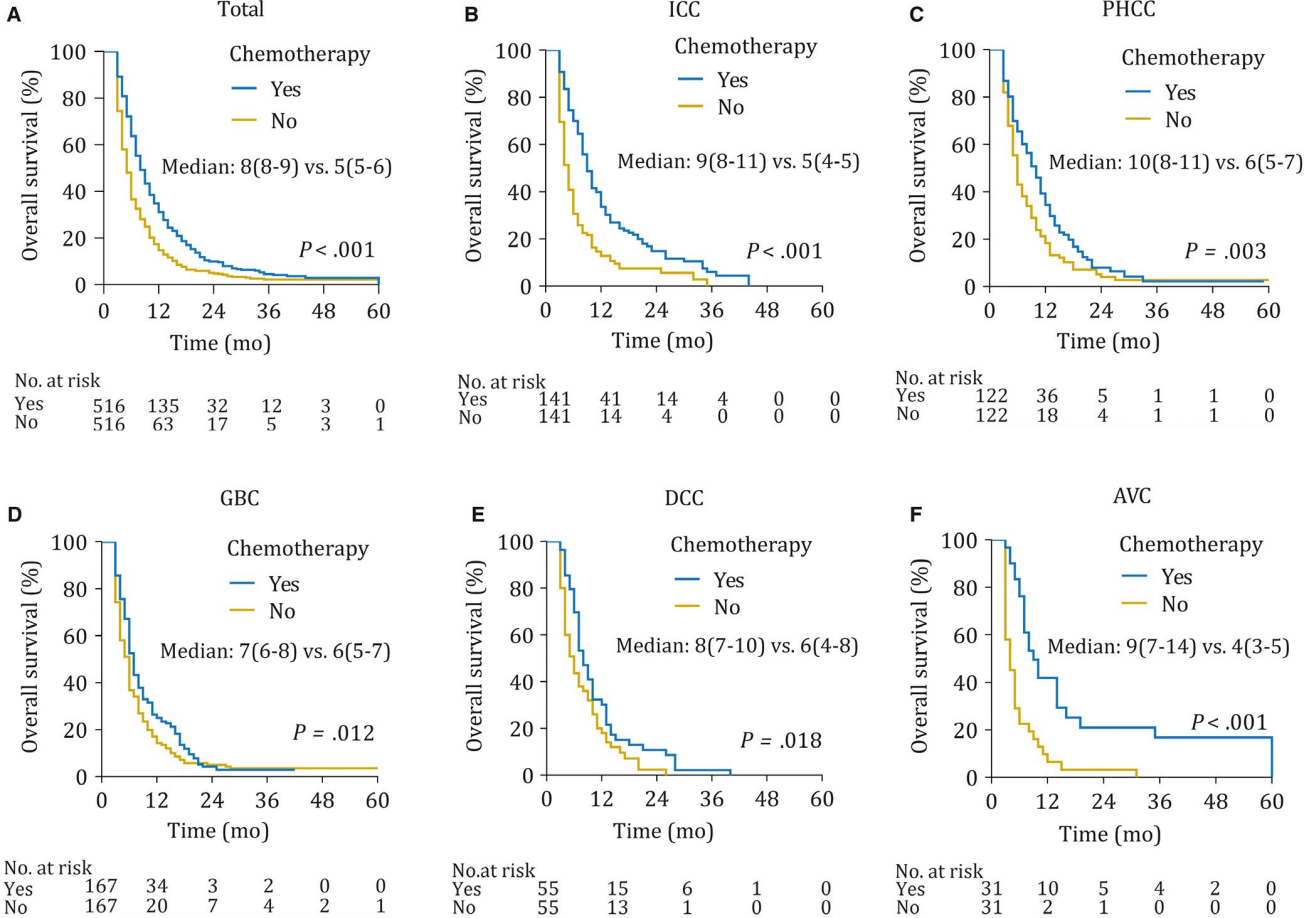
Kaplan‐Meier curves of overall survival according to whether or not receiving chemotherapy in each subtype of the BTC. AVC, Carcinoma of Vater ampulla; DCC, Distal cholangiocarcinoma; GBC, Gallbladder cancer; ICC, Intrahepatic cholangiocarcinoma; PHCC, Perihilar cholangiocarcinoma

Survival analysis was carried out subsequently, and the results are presented in Figure 4. In the total cohort, chemotherapy was significantly associated with improved survival months (chemotherapy vs no‐chemotherapy: 8, 95%CI: 8‐9 vs 5, 95%CI: 5‐6 for median OS) (*P* < .001). The improvement on OS after receiving chemotherapy also existed in each subtype (all *P* < .001); however, the level of survival improvement manifested different among the subtypes. The median OS for the ICC, PHCC, and AVC were elevated from 5 months to 9 months, from 6 months to 10 months, and from 4 months to 9 months after receiving chemotherapy, respectively. While, the elevation of survival time for the GBC and the DCC was only from 6 months to 7 months and from 6 months to 8 months, respectively, demonstrating insufficient efficacy of current chemotherapy regimens for the GBC and the DCC.

## 4 DISCUSSION

Correct understanding for the DM mode of the advanced BTC is helpful for accurate diagnosis and optimal treatment selection. Meanwhile, in consideration of the fact that clinical researches about treatment strategies in the BTC always included multiple subtypes, a valid and credible evaluation to the efficacy of current therapy in each subtype is urgently needed. In this study, we described the DM modes of the BTC in a large population, revealed the survival differences among DM sites, and found the different efficacy of current treatments in each subtype.

The DM mode of each subtype was apparently unique. Although liver only and dLN only were the top two metastasis sites in all the subtypes, DM rates of these subtypes extremely varied, ranging from 12.7% to 41.0%, and the proportions of the metastasis sites differed distinctively. About the differences of DM modes, there were some theoretical bases in clinical features and anatomy. Obstructive jaundice might develop at an early stage in the DCC and the AVC, while the GBC and the PHCC are always symptom‐free or just manifested by some nonspecific symptoms, such as sour regurgitation, nausea, and abdominal pain.^9^ Pathological difference might be an important factor, because the GBC and the PHCC are always more malignant and have a higher recurrence rate and a higher mortality rate after resection. Adjacency to visceral organ and complex vascular lymphatic system might also play an important role. The underlying mechanism is complicated and requires further investigations.

Despite the poor prognosis of the advanced BTC in whole, these metastasis sites still manifested different survival. Multi‐metastasis always indicated the shortest survival in our study, and dLN only always indicated a better prognosis, which might suggest the different disease progression, although they were both in M1 stage. Also, some metastasis sites showed different survival among the subtypes, which indicated the peculiarity of the DM mode of each subtype. Besides metastasis sites, there were some other clinic‐pathological factors, economic factors, and treatments relating to the patient survival. Most of the factors have been proved to influence the prognosis of the resectable BTC patients before, such as marital status, differentiation, and treatment, but the influence of T stage and N stage in the advanced BTC was fairly limited and not clearly as that in resectable cases.^10^, ^11^, ^12^ Interestingly, there is no factor having a significant survival effect in all the five subtypes except treatments. Choosing a proper treatment seemed the only thing that we could do for the advanced BTC patients, and a valid and credible evaluation to the efficacy of different therapies is of great value.

Radiation and palliative surgery did not work in the BTC according to our study, the previous researches, and the NCCN guideline.^13^ The remaining choice for M1 BTC patients was chemotherapy. As the gold standard, GemCis regimen (gemcitabine‐cisplatin) could elevate the median survival of the unresectable BTC patients from 8.1 to 11.7 months compared with gemcitabine monotherapy and did not reduce the quality of life.^14^, ^15^ A similar magnitude of benefit from the GemCis regimen was seen in the BT‐22 study.^16^ Besides, the GEMOX (gemcitabine‐oxaliplatin) also proved to be active and well tolerated in the unresectable BTC.^17^ However, these clinical researches included all the subtypes regardless of the different pathological basis, which severely limited the clinical application. In spite of the unavailability of the detailed regimes of chemotherapy in our study, improvement on survival after receiving chemotherapy also existed in each subtype. However, the level of survival improvement manifested different among the subtypes. In the PSM analysis, the median OS of patients receiving chemotherapy was nearly two times longer than those not receiving chemotherapy in the ICC, PHCC, and AVC, but the elevation of survival time for the GBC and the DCC was no more than 2 months. In view of the insufficient efficacy of chemotherapy, the role of targeted therapy and the immunotherapy needs further study. Targeted therapy and the immunotherapy showed effective results in some cancers, but the effect of them on the advanced BTC is still obscure. The effect of the EGFR inhibitors, the HER‐2 inhibitors, and the VEGF inhibitors did not demonstrate a better prognosis compared with GEMOX regimen, but part of patients could get a complete or partial remission.^18^, ^19^, ^20^, ^21^, ^22^ Immunotherapy might be a promising treatment for the advanced BTC because the expression of PD‐1/PD‐L1 elevated in the BTC tissue. Further researches are being carried out all over the world.^23^, ^24^

This study has some inevitable limitations primarily caused by the nature of the SEER database. First, the study was limited by its retrospective nature. It is necessary to validate our results in a prospective study or another large‐volume database. Second, there were still potential bias caused by the lack of some clinical characteristics, including palliative surgical procedures and the data regarding the detailed regimes of chemotherapy and radiation. Third, subgroup analyses for the chemotherapy efficacy of different metastasis sites in each subtype were not conducted for the insufficient patient numbers. At last, the record of peritoneal carcinomatosis was unavailable in SEER database, which is one of the common metastatic sites by BTC. Given these limitations, more efforts should be undertaken to validate our conclusion prior to clinical application.

## 5 CONCLUSION

In conclusion, we provided a detailed landscape of the DM mode of the advanced BTC in a large population, found the survival differences among DM sites, and revealed the different chemotherapy efficacy of the BTC subtypes.

## CONFLICT OF INTEREST

The authors declare no potential conflict of interest.

## AUTHOR CONTRIBUTIONS

J. Wang, X. Bo and L. Nan for acquisition of data, analysis and interpretation of data, statistical analysis and drafting of the manuscript; C. Wang, Z. Gao, T. Suo, X. Ni, and H. Liu for technical and material support; H. Liu, Y. Wang, and P. Lu for study concept and design, analysis and interpretation of data, drafting of the manuscript, obtained funding and study supervision. All authors read and approved the final manuscript.

## Data Availability

The raw data that generated cohort 1 and cohort 2 in this study are available upon request in the SEER (https://seer.cancer.gov/). The detailed information about metastasis mode and baseline characteristics of each subtype before and after PSM is available as Supporting Information.
