# Supplementary material for: Landscape of distant metastasis mode and current chemotherapy efficacy of the advanced biliary tract cancer in the United States, 2010‐2016
Source: Cancer Med. 2019 Dec 26;9(4):1335–48. doi: 10.1002/cam4.2794 (PMC7013071; doi:10.1002/cam4.2794)
Supplement: Supplementary file 1 [file CAM4-9-1335-s001.docx]

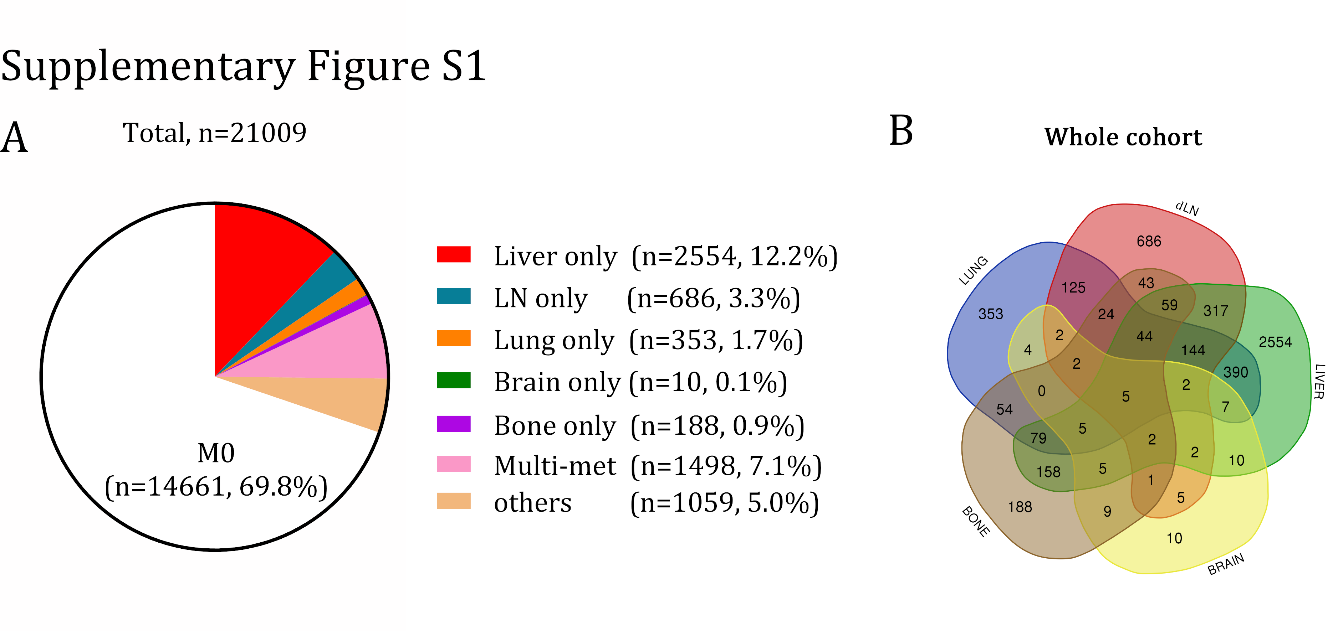


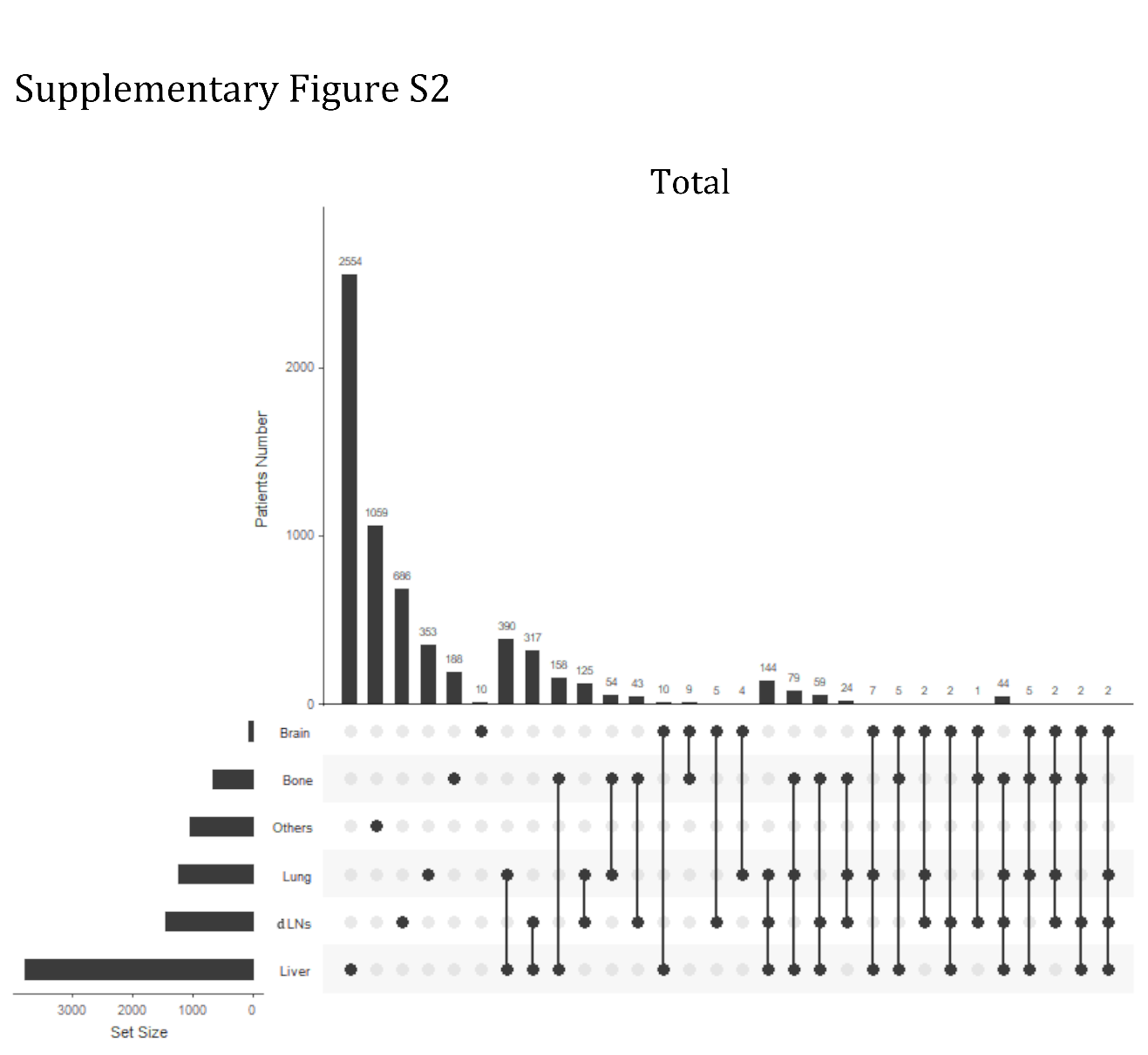


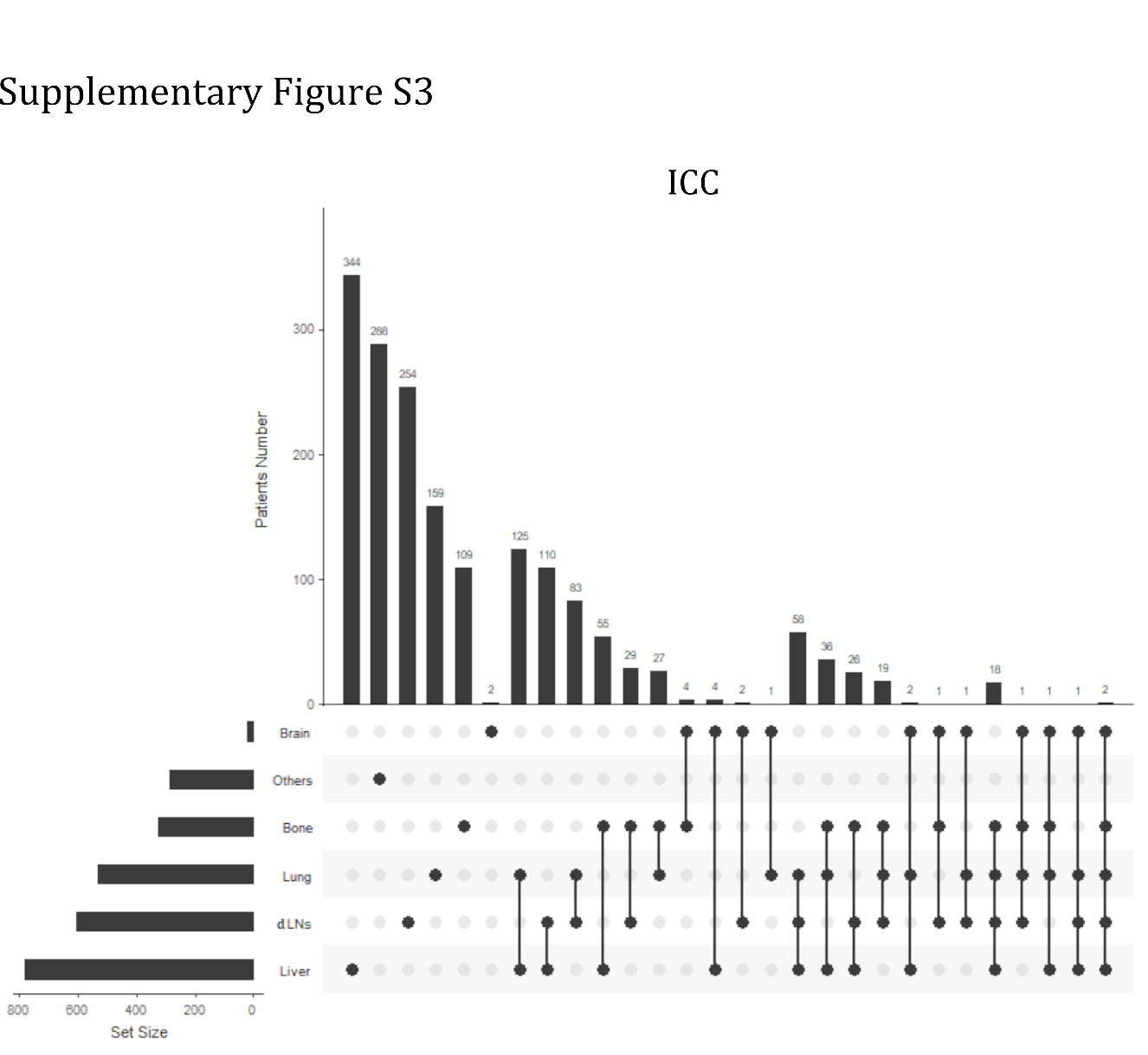


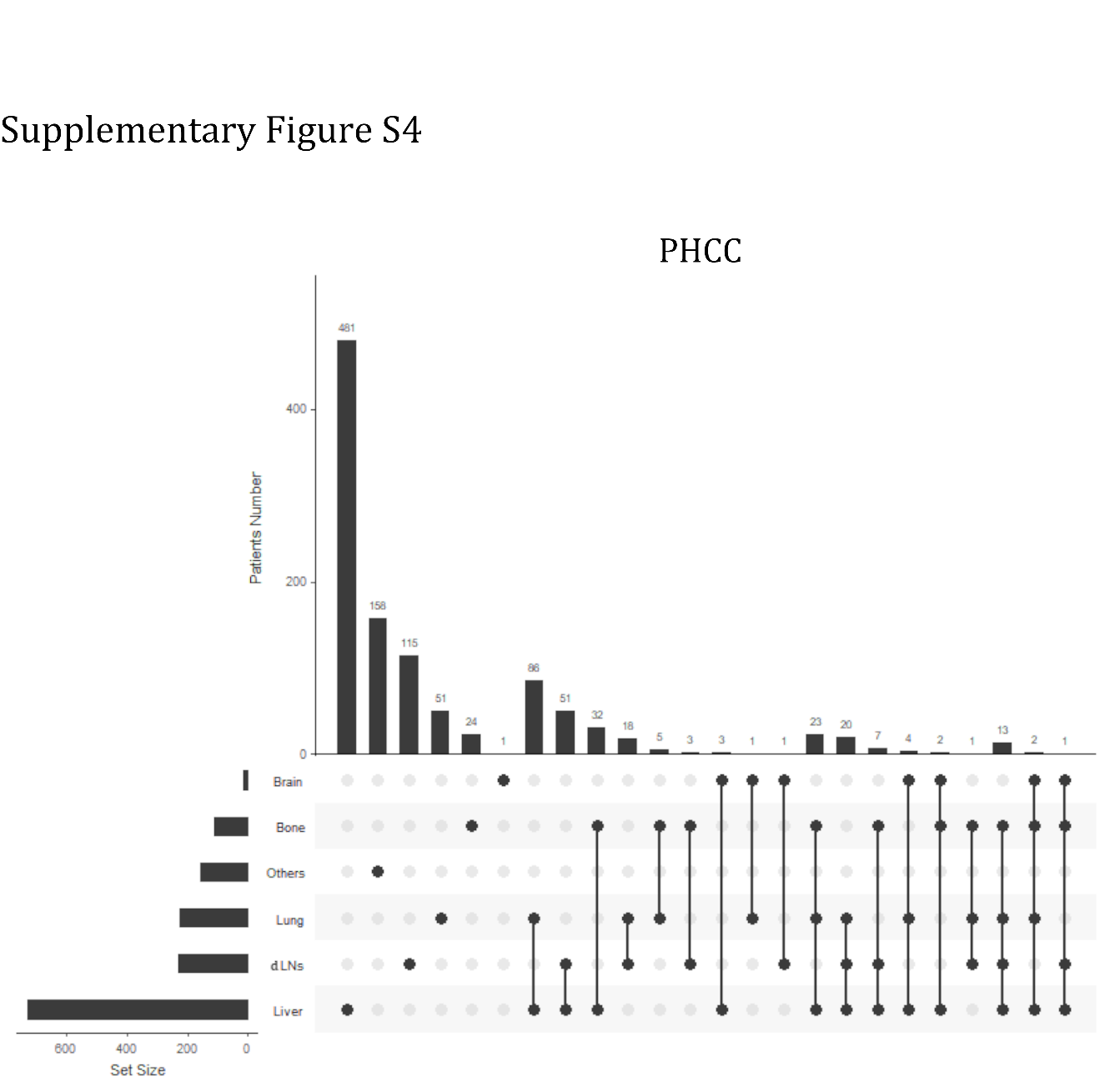


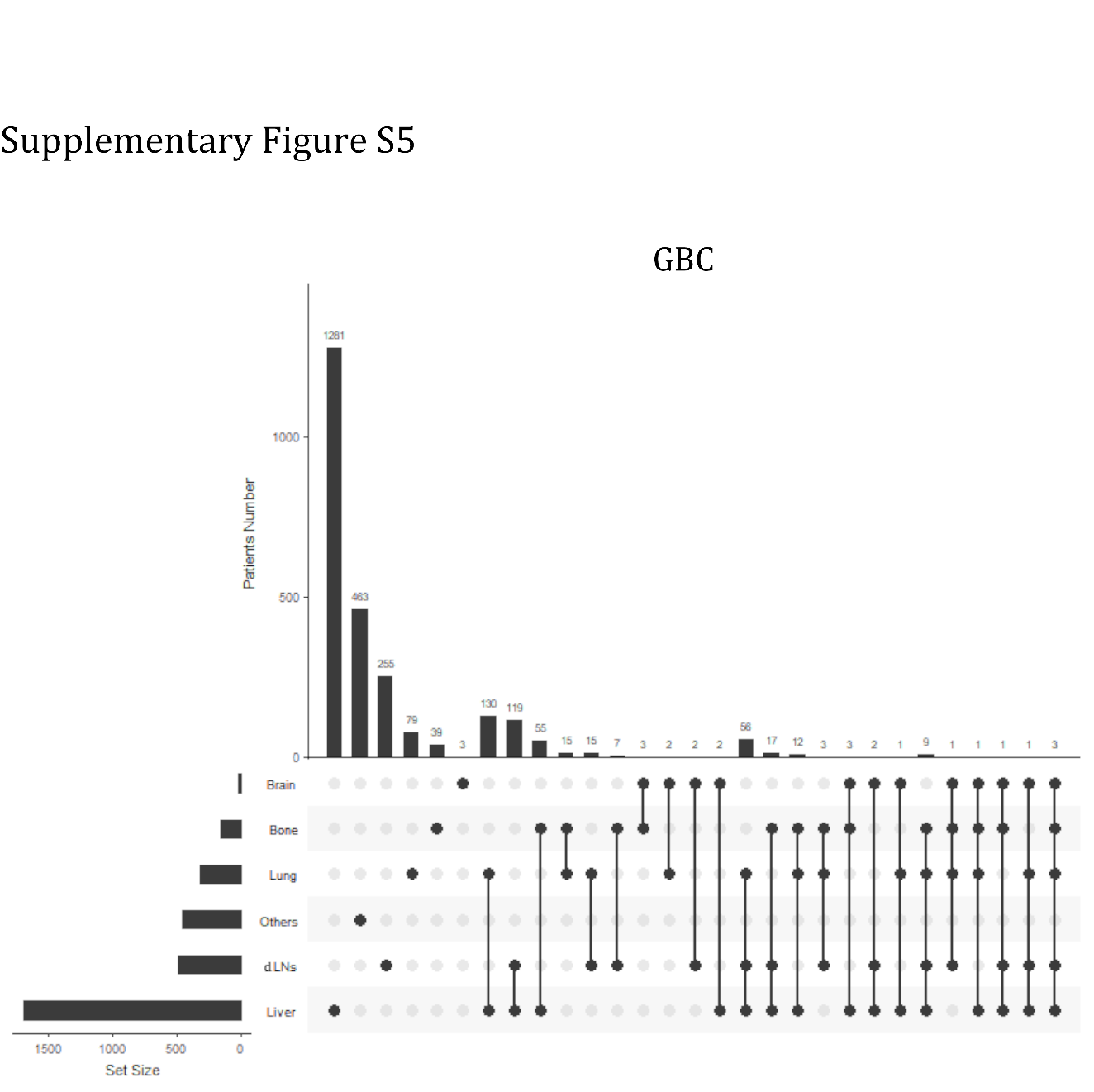


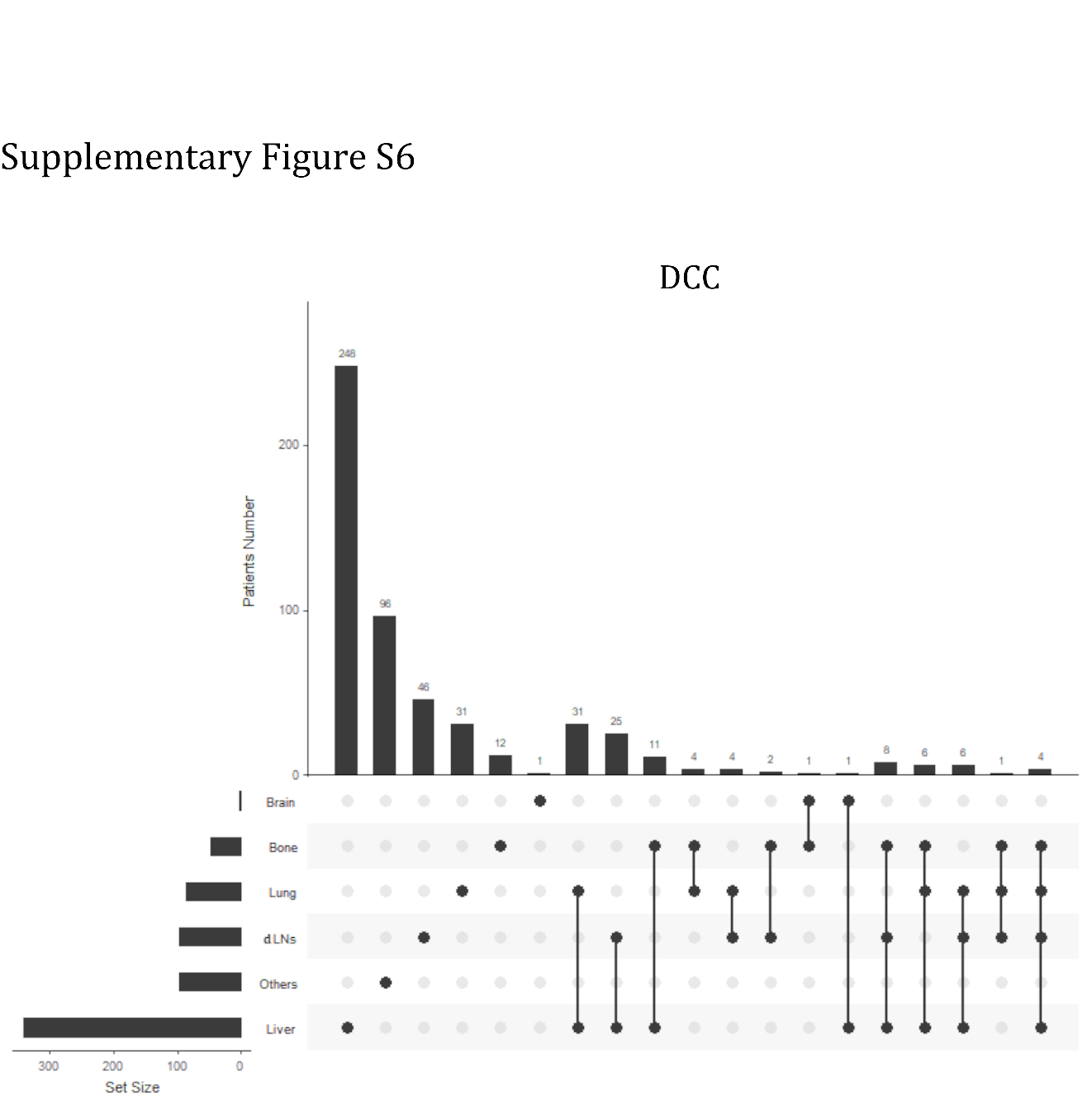


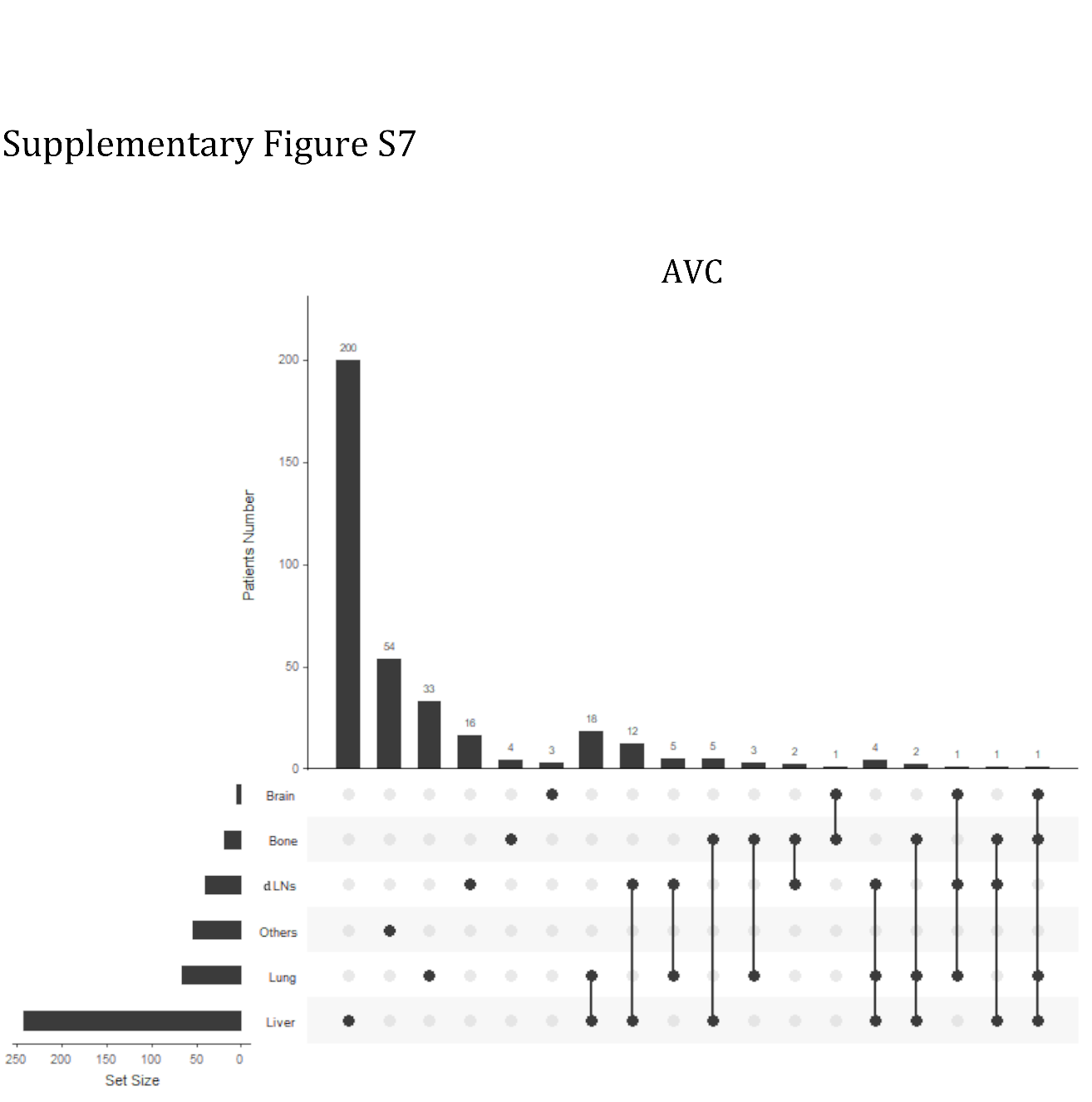


| Table S1. Univariate cox regression models of prognostic factors for overall survival | | | | | | | | | | | | | | | |
| --- | --- | --- | --- | --- | --- | --- | --- | --- | --- | --- | --- | --- | --- | --- | --- |
| Factors |  | ICC (n=1762) | |  | PHCC (n=1103) | |  | GBC (n=2580) | |  | DBDC (n=538) | |  | AVC (n=365) | |
|  |  | HR (95% CI) | *P* |  | HR (95% CI) | *P* |  | HR (95% CI) | *P* |  | HR (95% CI) | *P* |  | HR (95% CI) | *P* |
| Age, years | 60-69 vs. ≤59 | 1.274(1.121-1.448) | **<0.001** |  | 1.241(1.051-1.465) | **0.011** |  | 1.080(0.963-1.212) | 0.191 |  | 0.978(0.766-1.248) | 0.857 |  | 1.010(0.722-1.413) | 0.956 |
|  | 70-79 vs. ≤59 | 1.605(1.398-1.842) | **<0.001** |  | 1.453(1.219-1.731) | **<0.001** |  | 1.346(1.198-1.512) | **<0.001** |  | 1.387(1.072-1.794) | **0.013** |  | 1.441(1.044-1.990) | **0.027** |
|  | ≥80 vs. ≤59 | 2.402(2.017-2.859) | **<0.001** |  | 1.923(1.576-2.346) | **<0.001** |  | 2.064(1.824-2.336) | **<0.001** |  | 2.016(1.519-2.676 | **<0.001** |  | 2.001(1.416-2.828) | **<0.001** |
| Sex | Male vs. Female | 0.833(0.752-0.922) | **<0.001** |  | 1.006(0.889-1.139) | 0.922 |  | 1.054(0.964-1.153) | 0.251 |  | 0.932(0.779-1.115) | 0.446 |  | 0.854(0.678-1.074) | 0.179 |
| Race | Black vs. White | 1.055(0.876-1.269) | 0.576 |  | 1.052(0.849-1.303) | 0.645 |  | 0.984(0.875-1.106) | 0.785 |  | 1.415(1.061-1.886) | **0.019** |  | 1.002(0.714-1.406) | 0.991 |
|  | Others vs. White | 1.031(0.892-1.190) | 0.684 |  | 0.799(0.663-0.963) | **0.019** |  | 1.010(0.887-1.151) | 0.876 |  | 0.780(0.604-1.059) | 0.121 |  | 0.712(0.475-1.068) | 0.102 |
| Year at diagnosis | 14-16vs.10-13 | 1.018(0.917-1.129) | 0.742 |  | 1.034(0.912-1.173) | 0.601 |  | 0.914(0.839-0.995) | **0.039** |  | 0.945(0.786-1.137) | 0.553 |  | 0.949(0.743-1.211) | 0.673 |
| Marriage | Yes vs. No | 1.253(1.130-1.390) | **<0.001** |  | 1.124(0.992-1.273) | 0.067 |  | 1.293(1.190-1.404) | **<0.001** |  | 1.396(1.166-1.672) | **<0.001** |  | 0.899(0.715-1.130) | 0.362 |
| Income | Low vs. High | 1.079(0.974-1.195) | 0.146 |  | 1.023(0.904-1.158) | 0.719 |  | 1.127(1.037-1.224) | **0.005** |  | 0.944(0.789-1.129) | 0.531 |  | 1.056(0.839-1.330) | 0.645 |
| Insurance | No vs. Yes | 1.507(1.152-1.971) | **0.003** |  | 1.079(0.808-1.440) | 0.609 |  | 1.206(1.011-1.438) | **0.039** |  | 0.891(0.576-1.380) | 0.608 |  | 0.944(0.553-1.612) | 0.834 |
| Region | Pacific Coast vs. East | 1.294(1.062-1.578) | **0.011** |  | 0.762(0.586-0.991) | **0.044** |  | 1.124(0.964-1.310) | 0.137 |  | 0.922(0.672-1.265) | 0.617 |  | 0.993(0.636-1.550) | 0.976 |
|  | Northern Plain vs. East | 1.096(0.981-1.225) | 0.106 |  | 0.939(0.820-1.076) | 0.368 |  | 1.002(0.916-1.096) | 0.966 |  | 0.938(0.774-1.138) | 0.519 |  | 1.078(0.838-1.386) | 0.561 |
|  | Alaska/Southwest vs. East | 1.011(0.785-1.302) | 0.935 |  | 1.080(0.748-1.560) | 0.682 |  | 1.147(0.944-1.392) | 0.169 |  | 0.992(0.621-1.584) | 0.972 |  | 1.051(0.577-1.912) | 0.872 |
| Residence city | Middle vs. Large | 1.077(0.951-1.220) | 0.246 |  | 1.018(0.850-1.219) | 0.847 |  | 1.004(0.903-1.116) | 0.945 |  | 0.994(0.793-1.246) | 0.959 |  | 1.140(0.852-1.526) | 0.381 |
|  | Small vs. Large | 1.070(0.925-1.238) | 0.363 |  | 0.974(0.837-1.133) | 0.733 |  | 1.136(1.013-1.273) | **0.029** |  | 0.902(0.715-1.138) | 0.386 |  | 0.905(0.655-1.251) | 0.547 |
| Tumor size, mm | 30-59 vs. ≤29 | 1.062(0.822-1.371) | 0.648 |  | 1.133(0.889-1.445) | 0.316 |  | 1.179(1.016-1.369) | **0.031** |  | 1.327(0.954-1.847) | 0.095 |  | 1.103(0.786-1.548) | 0.573 |
|  | >60 vs. ≤29 | 1.082(0.862-1.358) | 0.499 |  | 1.193(0.909-1.566) | 0.205 |  | 1.380(1.181-1.613) | **<0.001** |  | 1.094(0.682-1.754) | 0.712 |  | 1.224(0.639-2.343) | 0.544 |
|  | Unknown vs. ≤29 | 1.347(1.080-1.679) | **0.009** |  | 1.367(1.143-1.636) | **<0.001** |  | 1.529(1.353-1.728) | **<0.001** |  | 1.476(1.171-1.860) | **0.001** |  | 1.232(0.949-1.598) | 0.119 |
| Differentiation | III/ IV vs. I-II | 1.276(1.059-1.536) | **0.011** |  | 1.090(0.831-1.431) | 0.535 |  | 1.449(1.276-1.645) | **<0.001** |  | 1.323(0.897-1.950) | 0.159 |  | 1.679(1.249-2.258) | **<0.001** |
|  | Unknown vs. I-II | 1.427(1.230-1.655) | **<0.001** |  | 1.201(0.970-1.488) | 0.095 |  | 1.765(1.576-1.976) | **<0.001** |  | 1.799(1.310-2.471) | **<0.001** |  | 1.379(1.050-1.811) | **0.022** |
| T stage | T3-T4 vs. T1-T2 | 0.947(0.827-1.084) | 0.428 |  | 0.999(0.819-1.219) | 0.994 |  | 1.191(1.066-1.330) | **0.002** |  | 0.982(0.777-1.240) | 0.881 |  | 0.888(0.681-1.157) | 0.381 |
|  | TX vs. T1-T2 | 1.173(1.041-1.322) | **0.009** |  | 1.090(0.949-1.252) | 0.227 |  | 1.555(1.376-1.757) | **<0.001** |  | 1.236(0.986-1.550) | 0.068 |  | 0.975(0.725-1.309) | 0.865 |
| N stage | N1 vs. N0 | 0.908(0.810-1.016) | 0.095 |  | 0.918(0.799-1.055) | 0.231 |  | 0.878(0.801-0.962) | **0.006** |  | 0.910(0.743-1.114) | 0.362 |  | 0.899(0.699-1.155) | 0.408 |
|  | Nx vs. N0 | 1.036(0.901-1.192) | 0.619 |  | 1.063(1.063-1.486) | **0.008** |  | 1.311(1.169-1.472) | **<0.001** |  | 1.123(0.873-1.445) | 0.369 |  | 1.027(0.736-1.433) | 0.875 |
| Metastasis site | Liver vs. dLN | 1.198(1.003-1.431) | **0.047** |  | 1.295(1.042-1.609) | **0.019** |  | 1.592(1.373-1.848) | **<0.001** |  | 1.200(0.851-1.691) | 0.301 |  | 1.109(0.603-2.039) | 0.742 |
|  | Lung vs. dLN | 1.194(0.963-1.480) | 0.108 |  | 1.383(0.982-1.949) | 0.065 |  | 1.267(0.962-1.670) | 0.094 |  | 1.231(0.753-2.012) | 0.411 |  | 1.117(0.554- 2.256) | 0.758 |
|  | Brain vs. dLN | 5.472(1.366-21.924) | **0.017** |  | 1.160(0.163-8.235) | 0.883 |  | 2.905(0.934-9.037) | 0.066 |  | 7.326(1.006-53.309) | 0.051 |  | 5.848(1.631-20.963) | **0.007** |
|  | Bone vs. dLN | 1.355(1.065-1.723) | **0.014** |  | 1.408(0.881-2.250) | 0.155 |  | 1.862(1.284-2.701) | **0.001** |  | 2.705(1.414-5.174) | **0.003** |  | 4.601(1.467-14.430) | **0.009** |
|  | Others vs. dLN | 1.122(0.933-1.349) | 0.225 |  | 1.143(0.887-1.473) | 0.304 |  | 1.386(1.173-1.639) | **<0.001** |  | 1.000(0.682-1.467) | 0.999 |  | 1.485(0.767-2.875) | 0.243 |
|  | Multi vs. dLN | 1.282(1.092-1.505) | **0.003** |  | 1.494(1.185-1.883) | **<0.001** |  | 1.748(1.477-2.069) | **<0.001** |  | 1.291(0.886-1.879) | 0.186 |  | 2.172(1.132-4.167) | **0.021** |
| Radiation | Yes vs. No | 0.778(0.660-0.916) | **0.003** |  | 0.683(0.542-0.862) | **0.001** |  | 0.659(0.561-0.775) | **<0.001** |  | 0.768(0.560-1.054) | 0.104 |  | 1.072(0.722-1.591) | 0.733 |
| Surgery | Yes vs. No | 0.528(0.401-0.695) | **<0.001** |  | 0.427(0.312-0.585) | **<0.001** |  | 0.594(0.543-0.650) | **<0.001** |  | 0.324(0.215-0.486) | **<0.001** |  | 0.486(0.337-0.702) | **<0.001** |
| Chemotherapy | Yes vs. No | 0.265(0.237-0.296) | **<0.001** |  | 0.411(0.362-0.468) | **<0.001** |  | 0.375(0.344-0.4083) | **<0.001** |  | 0.325(0.268-0.393) | **<0.001** |  | 0.356(0.282-0.450) | **<0.001** |
| ICC, Intrahepatic cholangiocarcinoma; PHCC, Perihilar cholangiocarcinoma; GBC, Gallbladder cancer; DBDC, Distal cholangiocarcinoma; AVC, Carcinoma of ampulla of Vater. dLN, Distant lymph node. HR, Hazard ratio. CI, Confidence interval. *P*<0.05 is considered statistically significant (bold). | | | | | | | | | | | | | | | |

| Table S2. Baseline characteristics of the without (No) and with chemotherapy (Yes) groups before and after PSM in ICC | | | | | | | | |
| --- | --- | --- | --- | --- | --- | --- | --- | --- |
|  |  | Before PSM | | |  | After PSM | | |
| Factors |  | No (n=155) | Yes (n=682) | *P* |  | No (n=141) | Yes (n=141) | *P* |
| Age, years | ≤59 | 37 | 277 | **<0.001** |  | 34 | 31 | 0.905 |
|  | 60-69 | 46 | 232 |  |  | 44 | 45 |  |
|  | 70-79 | 43 | 140 |  |  | 40 | 45 |  |
|  | ≥80 | 29 | 33 |  |  | 23 | 20 |  |
| Sex | Male | 76 | 354 | 0.577 |  | 71 | 73 | 0.905 |
|  | Female | 79 | 328 |  |  | 70 | 68 |  |
| Race | White | 113 | 531 | **0.168** |  | 104 | 111 | 0.579 |
|  | Black | 14 | 55 |  |  | 14 | 7 |  |
|  | Others | 28 | 96 |  |  | 23 | 23 |  |
| Year at diagnosis | 2010-1013 | 81 | 319 | **0.252** |  | 73 | 68 | 0.634 |
|  | 2014-2016 | 74 | 363 |  |  | 68 | 73 |  |
| Marriage | Yes | 78 | 437 | **0.002** |  | 76 | 81 | 0.632 |
|  | No | 77 | 245 |  |  | 65 | 60 |  |
| Income | Low income | 82 | 381 | 0.562 |  | 74 | 74 | 0.905 |
|  | High income | 73 | 301 |  |  | 67 | 67 |  |
| Insurance | Yes | 143 | 674 | **<0.001** |  | 2 | 4 | 0.679 |
|  | No | 12 | 8 |  |  | 139 | 137 |  |
| Region | East | 46 | 252 | 0.055 |  | 40 | 50 | 0.587 |
|  | Pacific Coast | 10 | 47 |  |  | 10 | 7 |  |
|  | Northern Plain | 90 | 357 |  |  | 83 | 77 |  |
|  | Alaska/Southwest | 9 | 26 |  |  | 8 | 7 |  |
| Residence city | Small | 102 | 443 | 0.869 |  | 94 | 83 | 0.353 |
|  | Middle | 33 | 140 |  |  | 29 | 33 |  |
|  | Large | 20 | 99 |  |  | 18 | 25 |  |
| Tumor size, mm | ≤30 | 10 | 49 | **0.038** |  | 9 | 15 | 0.623 |
|  | 30-59 | 24 | 88 |  |  | 23 | 15 |  |
|  | >60 | 37 | 242 |  |  | 36 | 42 |  |
|  | Unknown | 84 | 303 |  |  | 73 | 69 |  |
| Differentiation | I-II | 25 | 115 | 0.521 |  | 24 | 20 | 0.751 |
|  | III-IV | 18 | 99 |  |  | 17 | 21 |  |
|  | Unknown | 112 | 468 |  |  | 100 | 100 |  |
| T stage | T1-T2 | 73 | 387 | **<0.001** |  | 70 | 76 | 0.499 |
|  | T3-T4 | 25 | 146 |  |  | 25 | 28 |  |
|  | TX | 57 | 149 |  |  | 46 | 37 |  |
| N stage | N0 | 62 | 287 | <0.001 |  | 59 | 66 | 0.550 |
|  | N1 | 50 | 292 |  |  | 48 | 48 |  |
|  | Nx | 43 | 103 |  |  | 34 | 27 |  |
| Metastasis site | Distant lymph node only | 25 | 117 | **0.006** |  | 24 | 29 | 0.878 |
|  | Liver only | 44 | 126 |  |  | 35 | 37 |  |
|  | Lung only | 14 | 68 |  |  | 13 | 9 |  |
|  | Brain only | 0 | 0 |  |  | 0 | 0 |  |
|  | Bone only | 8 | 27 |  |  | 7 | 4 |  |
|  | Other | 34 | 117 |  |  | 32 | 22 |  |
|  | Multi-metastasis | 30 | 227 |  |  | 30 | 40 |  |
| ICC, Intrahepatic cholangiocarcinoma. P<0.05 is considered statistically significant (bold). | | | | | | | | |

| Table S3. Baseline characteristics of the without (No) and with chemotherapy (Yes) groups before and after PSM in PHCC | | | | | | | | |
| --- | --- | --- | --- | --- | --- | --- | --- | --- |
|  |  | Before PSM | | |  | After PSM | | |
| Factors |  | No (n=145) | Yes (n=356) | *P* |  | No (n=122) | Yes (n=122) | *P* |
| Age, years | ≤59 | 31 | 119 | **<0.001** |  | 31 | 28 | 0.266 |
|  | 60-69 | 35 | 132 |  |  | 34 | 43 |  |
|  | 70-79 | 35 | 88 |  |  | 28 | 37 |  |
|  | ≥80 | 44 | 17 |  |  | 29 | 14 |  |
| Sex | Male | 60 | 174 | 0.154 |  | 58 | 60 | 0.898 |
|  | Female | 85 | 182 |  |  | 64 | 62 |  |
| Race | White | 100 | 288 | **0.001** |  | 89 | 87 | 0.707 |
|  | Black | 15 | 31 |  |  | 12 | 16 |  |
|  | Others | 30 | 37 |  |  | 21 | 19 |  |
| Year at diagnosis | 2010-1013 | 79 | 193 | **0.965** |  | 69 | 63 | 0.521 |
|  | 2014-2016 | 66 | 163 |  |  | 53 | 59 |  |
| Marriage | Yes | 61 | 218 | **<0.001** |  | 57 | 57 | 0.898 |
|  | No | 84 | 138 |  |  | 65 | 65 |  |
| Income | Low income | 62 | 168 | 0.421 |  | 53 | 56 | 0.797 |
|  | High income | 83 | 188 |  |  | 69 | 66 |  |
| Insurance | Yes | 7 | 17 | **0.837** |  | 6 | 7 | 1.000 |
|  | No | 138 | 339 |  |  | 116 | 115 |  |
| Region | East | 34 | 122 | **0.022** |  | 29 | 43 | 0.224 |
|  | Pacific Coast | 13 | 29 |  |  | 10 | 10 |  |
|  | Northern Plain | 94 | 197 |  |  | 79 | 67 |  |
|  | Alaska/Southwest | 4 | 8 |  |  | 4 | 2 |  |
| Residence city | Small | 100 | 218 | **0.028** |  | 85 | 74 | 0.097 |
|  | Middle | 33 | 80 |  |  | 27 | 27 |  |
|  | Large | 12 | 58 |  |  | 10 | 21 |  |
| Tumor size, mm | ≤30 | 26 | 54 | 0.238 |  | 20 | 18 | 0.767 |
|  | 30-59 | 23 | 45 |  |  | 17 | 16 |  |
|  | >60 | 6 | 31 |  |  | 6 | 10 |  |
|  | Unknown | 90 | 226 |  |  | 79 | 78 |  |
| Differentiation | I-II | 21 | 28 | 0.030 |  | 16 | 10 | 0.696 |
|  | III-IV | 11 | 45 |  |  | 9 | 17 |  |
|  | Unknown | 113 | 283 |  |  | 97 | 95 |  |
| T stage | T1-T2 | 46 | 107 | **0.634** |  | 39 | 32 | 0.612 |
|  | T3-T4 | 18 | 56 |  |  | 18 | 19 |  |
|  | TX | 81 | 193 |  |  | 65 | 71 |  |
| N stage | N0 | 63 | 153 | 0.989 |  | 54 | 55 | 0.944 |
|  | N1 | 56 | 140 |  |  | 46 | 47 |  |
|  | Nx | 26 | 63 |  |  | 22 | 20 |  |
| Metastasis site | Distant lymph node only | 20 | 40 | **0.020** |  | 15 | 10 | 0.859 |
|  | Liver only | 66 | 145 |  |  | 53 | 64 |  |
|  | Lung only | 11 | 13 |  |  | 9 | 4 |  |
|  | Brain only | 0 | 0 |  |  | 0 | 0 |  |
|  | Bone only | 4 | 5 |  |  | 4 | 1 |  |
|  | Other | 20 | 62 |  |  | 19 | 16 |  |
|  | Multi-metastasis | 24 | 91 |  |  | 22 | 27 |  |
| PHCC, Perihilar cholangiocarcinoma. P<0.05 is considered statistically significant (bold). | | | | | | | | |

| Table S4. Baseline characteristics of the without (No) and with chemotherapy (Yes) groups before and after PSM in GBC | | | | | | | | |
| --- | --- | --- | --- | --- | --- | --- | --- | --- |
|  |  | Before PSM | | |  | After PSM | | |
|  |  | No (n=197) | Yes (n=567) | *P* |  | No (n=167) | Yes (n=167) | *P* |
| Age, years | ≤59 | 41 | 191 | **<0.001** |  | 41 | 34 | 0.483 |
|  | 60-69 | 44 | 187 |  |  | 44 | 52 |  |
|  | 70-79 | 50 | 139 |  |  | 47 | 53 |  |
|  | ≥80 | 62 | 50 |  |  | 35 | 28 |  |
| Sex | Male | 60 | 183 | 0.702 |  | 53 | 55 | 0.907 |
|  | Female | 137 | 384 |  |  | 114 | 112 |  |
| Race | White | 139 | 397 | **0.059** |  | 118 | 116 | 0.703 |
|  | Black | 24 | 101 |  |  | 23 | 32 |  |
|  | Others | 34 | 69 |  |  | 26 | 19 |  |
| Year at diagnosis | 2010-1013 | 118 | 315 | **0.329** |  | 100 | 104 | 0.736 |
|  | 2014-2016 | 79 | 252 |  |  | 67 | 63 |  |
| Marriage | Yes | 70 | 341 | **<0.001** |  | 68 | 75 | 0.507 |
|  | No | 127 | 226 |  |  | 99 | 92 |  |
| Income | Low income | 102 | 291 | 0.978 |  | 84 | 85 | 1.000 |
|  | High income | 95 | 276 |  |  | 83 | 82 |  |
| Insurance | Yes | 11 | 23 | **0.487** |  | 8 | 12 | 0.489 |
|  | No | 186 | 544 |  |  | 159 | 155 |  |
| Region | East | 64 | 238 | **0.007** |  | 57 | 70 | 0.166 |
|  | Pacific Coast | 13 | 48 |  |  | 9 | 14 |  |
|  | Northern Plain | 107 | 254 |  |  | 91 | 78 |  |
|  | Alaska/Southwest | 13 | 27 |  |  | 10 | 5 |  |
| Residence city | Small | 135 | 369 | 0.175 |  | 114 | 110 | 0.389 |
|  | Middle | 32 | 126 |  |  | 27 | 36 |  |
|  | Large | 30 | 72 |  |  | 26 | 21 |  |
| Tumor size, mm | ≤30 | 22 | 64 | 0.915 |  | 20 | 24 | 0.921 |
|  | 30-59 | 32 | 95 |  |  | 27 | 24 |  |
|  | >60 | 29 | 81 |  |  | 25 | 21 |  |
|  | Unknown | 114 | 327 |  |  | 95 | 98 |  |
| Differentiation | I-II | 18 | 64 | 0.132 |  | 18 | 16 | 1.000 |
|  | III-IV | 23 | 89 |  |  | 20 | 24 |  |
|  | Unknown | 156 | 414 |  |  | 129 | 127 |  |
| T stage | T1-T2 | 21 | 70 | **0.690** |  | 19 | 22 | 0.669 |
|  | T3-T4 | 100 | 295 |  |  | 87 | 79 |  |
|  | TX | 76 | 202 |  |  | 61 | 66 |  |
| N stage | N0 | 80 | 198 | <0.001 |  | 74 | 61 | 0.528 |
|  | N1 | 64 | 269 |  |  | 52 | 69 |  |
|  | Nx | 53 | 100 |  |  | 41 | 37 |  |
| Metastasis site | Distant lymph node only | 14 | 62 | **<0.001** |  | 11 | 22 | 0.790 |
|  | Liver only | 111 | 248 |  |  | 89 | 73 |  |
|  | Lung only | 8 | 26 |  |  | 5 | 9 |  |
|  | Brain only | 1 | 0 |  |  | 1 | 0 |  |
|  | Bone only | 2 | 5 |  |  | 2 | 0 |  |
|  | Other | 35 | 86 |  |  | 33 | 27 |  |
|  | Multi-metastasis | 26 | 140 |  |  | 26 | 36 |  |
| GBC, Gallbladder cancer. P<0.05 is considered statistically significant (bold). | | | | | | | | |

| Table S5. Baseline characteristics of the without (No) and with chemotherapy (Yes) groups before and after PSM in DCC | | | | | | | | |
| --- | --- | --- | --- | --- | --- | --- | --- | --- |
|  |  | Before PSM | | |  | After PSM | | |
| Factors |  | No (n=79) | Yes (n=165) | *P* |  | No (n=55) | Yes (n=55) | *P* |
| Age, years | ≤59 | 15 | 48 | **0.002** |  | 11 | 13 | 0.177 |
|  | 60-69 | 26 | 61 |  |  | 23 | 18 |  |
|  | 70-79 | 18 | 43 |  |  | 13 | 21 |  |
|  | ≥80 | 20 | 13 |  |  | 8 | 3 |  |
| Sex | Male | 39 | 75 | 0.663 |  | 29 | 34 | 0.441 |
|  | Female | 40 | 90 |  |  | 26 | 21 |  |
| Race | White | 57 | 127 | **0.539** |  | 42 | 41 | 0.887 |
|  | Black | 12 | 17 |  |  | 6 | 9 |  |
|  | Others | 10 | 21 |  |  | 7 | 5 |  |
| Year at diagnosis | 2010-1013 | 46 | 93 | **0.891** |  | 32 | 30 | 0.848 |
|  | 2014-2016 | 33 | 72 |  |  | 23 | 25 |  |
| Marriage | Yes | 38 | 99 | **0.106** |  | 30 | 30 | 0.848 |
|  | No | 41 | 66 |  |  | 25 | 25 |  |
| Income | Low income | 38 | 81 | 0.994 |  | 30 | 24 | 0.340 |
|  | High income | 41 | 84 |  |  | 25 | 31 |  |
| Insurance | Yes | 5 | 4 | **0.249** |  | 0 | 1 | 1.000 |
|  | No | 74 | 161 |  |  | 55 | 54 |  |
| Region | East | 30 | 80 | 0.177 |  | 21 | 30 | 0.189 |
|  | Pacific Coast | 10 | 16 |  |  | 5 | 3 |  |
|  | Northern Plain | 37 | 65 |  |  | 28 | 19 |  |
|  | Alaska/Southwest | 2 | 4 |  |  | 1 | 3 |  |
| Residence city | Small | 51 | 91 | 0.176 |  | 36 | 28 | 0.297 |
|  | Middle | 15 | 38 |  |  | 10 | 15 |  |
|  | Large | 13 | 36 |  |  | 9 | 12 |  |
| Tumor size, mm | ≤30 | 14 | 37 | 0.544 |  | 12 | 12 | 0.554 |
|  | 30-59 | 9 | 23 |  |  | 6 | 7 |  |
|  | >60 | 3 | 10 |  |  | 2 | 0 |  |
|  | Unknown | 53 | 95 |  |  | 35 | 36 |  |
| Differentiation | I-II | 8 | 15 | 0.803 |  | 6 | 4 | 0.764 |
|  | III-IV | 10 | 26 |  |  | 5 | 11 |  |
|  | Unknown | 61 | 124 |  |  | 44 | 40 |  |
| T stage | T1-T2 | 24 | 42 | **0.340** |  | 17 | 15 | 0.732 |
|  | T3-T4 | 20 | 57 |  |  | 12 | 19 |  |
|  | TX | 35 | 66 |  |  | 26 | 21 |  |
| N stage | N0 | 44 | 80 | 0.115 |  | 34 | 30 | 0.681 |
|  | N1 | 19 | 61 |  |  | 14 | 19 |  |
|  | Nx | 16 | 24 |  |  | 7 | 6 |  |
| Metastasis site | Distant lymph node only | 7 | 15 | **0.287** |  | 4 | 6 | 0.169 |
|  | Liver only | 40 | 74 |  |  | 31 | 24 |  |
|  | Lung only | 6 | 12 |  |  | 3 | 1 |  |
|  | Brain only | 0 | 0 |  |  | 0 | 0 |  |
|  | Bone only | 1 | 0 |  |  | 0 | 0 |  |
|  | Other | 17 | 30 |  |  | 12 | 10 |  |
|  | Multi-metastasis | 8 | 34 |  |  | 5 | 14 |  |
| DCC, Distal cholangiocarcinoma. P<0.05 is considered statistically significant (bold). | | | | | | | | |

| Table S6. Baseline characteristics of the without (No) and with chemotherapy (Yes) groups before and after PSM in AVC. | | | | | | | | |
| --- | --- | --- | --- | --- | --- | --- | --- | --- |
|  |  | Before PSM | | |  | After PSM | | |
| Factors |  | No (n=55) | Yes (n=136) | *P* |  | No (n=31) | Yes (n=31) | *P* |
| Age, years | ≤59 | 8 | 40 | **<0.001** |  | 8 | 6 | 0.898 |
|  | 60-69 | 12 | 43 |  |  | 9 | 11 |  |
|  | 70-79 | 13 | 41 |  |  | 8 | 11 |  |
|  | ≥80 | 22 | 12 |  |  | 6 | 3 |  |
| Sex | Male | 27 | 70 | 0.890 |  | 15 | 18 | 0.611 |
|  | Female | 28 | 66 |  |  | 16 | 13 |  |
| Race | White | 46 | 110 | **0.529** |  | 26 | 24 | 0.809 |
|  | Black | 7 | 15 |  |  | 3 | 4 |  |
|  | Others | 2 | 11 |  |  | 2 | 3 |  |
| Year at diagnosis | 2010-1013 | 37 | 74 | **0.142** |  | 19 | 21 | 0.791 |
|  | 2014-2016 | 18 | 62 |  |  | 12 | 10 |  |
| Marriage | Yes | 19 | 77 | **0.009** |  | 15 | 16 | 1.000 |
|  | No | 36 | 59 |  |  | 16 | 15 |  |
| Income | Low income | 24 | 69 | 0.466 |  | 14 | 17 | 0.612 |
|  | High income | 31 | 67 |  |  | 17 | 14 |  |
| Insurance | Yes | 2 | 3 | **0.952** |  | 1 | 1 | 0.472 |
|  | No | 53 | 133 |  |  | 30 | 30 |  |
| Region | East | 16 | 46 | 0.173 |  | 8 | 12 | 0.412 |
|  | Pacific Coast | 2 | 12 |  |  | 1 | 2 |  |
|  | Northern Plain | 37 | 73 |  |  | 22 | 17 |  |
|  | Alaska/Southwest | 0 | 5 |  |  | 0 | 0 |  |
| Residence city | Small | 32 | 93 | **0.048** |  | 19 | 25 | 0.246 |
|  | Middle | 16 | 19 |  |  | 9 | 3 |  |
|  | Large | 7 | 24 |  |  | 3 | 3 |  |
| Tumor size, mm | ≤30 | 18 | 43 | 0.284 |  | 11 | 8 | 0.851 |
|  | 30-59 | 5 | 21 |  |  | 3 | 5 |  |
|  | >60 | 0 | 5 |  |  | 0 | 3 |  |
|  | Unknown | 32 | 67 |  |  | 17 | 15 |  |
| Differentiation | I-II | 20 | 49 | 0.848 |  | 10 | 15 | 0.395 |
|  | III-IV | 14 | 30 |  |  | 8 | 5 |  |
|  | Unknown | 21 | 57 |  |  | 13 | 11 |  |
| T stage | T1-T2 | 22 | 55 | **0.623** |  | 12 | 13 | 0.959 |
|  | T3-T4 | 20 | 41 |  |  | 12 | 11 |  |
|  | TX | 13 | 40 |  |  | 7 | 7 |  |
| N stage | N0 | 35 | 68 | 0.110 |  | 17 | 17 | 1.000 |
|  | N1 | 10 | 45 |  |  | 8 | 8 |  |
|  | Nx | 10 | 23 |  |  | 6 | 6 |  |
| Metastasis site | Distant lymph node only | 2 | 9 | **0.203** |  | 2 | 1 | 0.896 |
|  | Liver only | 33 | 84 |  |  | 19 | 19 |  |
|  | Lung only | 5 | 13 |  |  | 3 | 3 |  |
|  | Brain only | 1 | 0 |  |  | 1 | 0 |  |
|  | Bone only | 0 | 0 |  |  | 0 | 0 |  |
|  | Other | 9 | 10 |  |  | 4 | 5 |  |
|  | Multi-metastasis | 5 | 20 |  |  | 2 | 3 |  |
| AVC, Carcinoma of ampulla of Vater P<0.05 is considered statistically significant (bold). | | | | | | | | |
